# Targeting Immune-Fibroblast Crosstalk in Myocardial Infarction and Cardiac Fibrosis

**DOI:** 10.21203/rs.3.rs-2402606/v1

**Published:** 2023-01-26

**Authors:** Junedh M. Amrute, Xin Luo, Vinay Penna, Andrea Bredemeyer, Tracy Yamawaki, Steven Yang, Farid Kadyrov, Gyu-Seong Heo, Sally Yu Shi, Paul Lee, Andrew L. Koenig, Christoph Kuppe, Cameran Jones, Benjamin Kopecky, Sikander Hayat, Pan Ma, Yuriko Terada, Angela Fu, Milena Furtado, Daniel Kreisel, Nathan O. Stitziel, Chi-Ming Li, Rafael Kramann, Yongjian Liu, Brandon Ason, Kory J. Lavine

**Affiliations:** 1 Center for Cardiovascular Research, Division of Cardiology, Department of Medicine, Washington University School of Medicine, Saint Louis, MO, 63110, USA; 2 Genome Analysis Unit, Amgen Discovery Research, Amgen Inc., 1120 Veterans Blvd, South San Francisco, CA, 94080, USA; 3 Department of Cardiometabolic Disorders, Amgen Discovery Research, Amgen Inc., 1120 Veterans Blvd, South San Francisco, CA, 94080, USA; 4 Mallinckrodt Institute of Radiology, Washington University School of Medicine, Saint Louis, MO, 63110, USA; 5 Institute of Experimental Medicine and Systems Biology, RWTH Aachen University, Medical Faculty, Aachen, Germany; 6 Department of Nephrology, RWTH Aachen, Medical Faculty, Aachen, Germany; 7 Division of Cardiothoracic Surgery, Department of Surgery, Washington University School of Medicine, Saint Louis, MO, 63110, USA; 8 Department of Pathology and Immunology, Washington University School of Medicine, Saint Louis, MO, 63110, USA; 9 Department of Genetics, Washington University School of Medicine, Saint Louis, MO, 63110, USA; 10 McDonnell Genome Institute, Washington University School of Medicine, Saint Louis, MO, 63110, USA; 11 Department of Internal Medicine, Nephrology and Transplantation Erasmus Medical Center, Rotterdam, The Netherlands; 12 Department of Developmental Biology, Washington University School of Medicine, Saint Louis, MO, 63110, USA

**Keywords:** heart failure, fibroblast activator protein, macrophages, C-C chemokine receptor 2, interleukin 1 beta, fibrosis

## Abstract

Inflammation and tissue fibrosis co-exist and are causally linked to organ dysfunction. However, the molecular mechanisms driving immune-fibroblast crosstalk in human cardiac disease remains unexplored and there are currently no therapeutics to target fibrosis. Here, we performed multi-omic single-cell gene expression, epitope mapping, and chromatin accessibility profiling in 38 donors, acutely infarcted, and chronically failing human hearts. We identified a disease-associated fibroblast trajectory marked by cell surface expression of fibroblast activator protein (FAP), which diverged into distinct myofibroblasts and pro-fibrotic fibroblast populations, the latter resembling matrifibrocytes. Pro-fibrotic fibroblasts were transcriptionally similar to cancer associated fibroblasts and expressed high levels of collagens and periostin (*POSTN*), thymocyte differentiation antigen 1 (THY-1), and endothelin receptor A (*EDNRA*) predicted to be driven by a *RUNX1* gene regulatory network. We assessed the applicability of experimental systems to model tissue fibrosis and demonstrated that 3 different *in vivo* mouse models of cardiac injury were superior compared to cultured human heart and dermal fibroblasts in recapitulating the human disease phenotype. Ligand-receptor analysis and spatial transcriptomics predicted that interactions between C-C chemokine receptor type 2 (CCR2) macrophages and fibroblasts mediated by interleukin 1 beta (IL-1β) signaling drove the emergence of pro-fibrotic fibroblasts within spatially defined niches. This concept was validated through *in silico* transcription factor perturbation and *in vivo* inhibition of IL-1β signaling in fibroblasts where we observed reduced pro-fibrotic fibroblasts, preferential differentiation of fibroblasts towards myofibroblasts, and reduced cardiac fibrosis. Herein, we show a subset of macrophages signal to fibroblasts via IL-1β and rewire their gene regulatory network and differentiation trajectory towards a pro-fibrotic fibroblast phenotype. These findings highlight the broader therapeutic potential of targeting inflammation to treat tissue fibrosis and restore organ function.

## Introduction

Tissue fibrosis is an important determinant of organ dysfunction with tremendous clinical impact. Today, there are no therapeutics to target fibrosis. Cardiac fibrosis contributes to life-threatening arrhythmias, heart failure progression, and mortality. While present in nearly all forms of heart disease, cardiac fibrosis is prominent in patients who suffer a myocardial infarction (MI) and those with both ischemic and non-ischemic cardiomyopathies, where fibrosis is associated with loss of viable myocardium^1,2^. Despite advancements in clinical management, MI and chronic cardiomyopathies are among the leading causes of heart failure, associated morbidity, and death worldwide^3,4^ and continue to impose staggering financial burden on healthcare systems^3,5–8^.

Strategies to therapeutically target cardiac fibrosis have remained elusive. Recent advances in CAR T-cell technology have provided a much needed breakthrough and indicated that selective removal of cardiac fibroblasts that express fibroblast activator protein (FAP) is sufficient to ameliorate fibrosis without impacting the structural integrity of the heart in pre-clinical mouse models^9,10^. While these studies suggested that targeted fibroblast subset depletion may be therapeutically achievable and challenged the dogma that cardiac fibrosis is irreversible, many questions remain to be answered. Numerous studies have elegantly characterized fibroblast subtypes in mouse models of cardiac injury^11–13^. However, the precise fibroblast populations that are responsible for fibrosis in the human heart and mechanisms that orchestrate their emergence and maturation are unknown. Furthermore, it is not clear which of the many available pre-clinical animal and *in vitro* experimental models ^14–19^ best recapitulate human cardiac fibrosis.

The advent of high-throughput sequencing technologies has enabled high resolution profiling of healthy and diseased tissues to discover novel cell types^20,21^. Numerous single-nucleus (snRNA-seq) studies have studied the healthy and failing human heart^22–26^. However, these studies are performed on frozen tissue and only include transcriptomic information which restrains high-resolution cell phenotyping. Furthermore, the reliance on only nuclear RNA precludes cellular transcriptomic, epigenetic, and proteomic profiling of diseased states. These studies lack the granularity to detect the precise immune and fibroblast populations which emerge after a myocardial infarction and drive cardiac fibrosis. Given the challenges associated with collecting fresh cardiac left ventricle tissue, there is no large-scale study which performs single cell or epitope-based sequencing in human hearts.

We performed Cellular Indexing of Transcriptomes and Epitomes by sequencing (CITE-seq)^27^ on freshly explanted human hearts and Multiomic (paired single nucleus RNA + assay for transposable accessible chromatin, ATAC) sequencing from non-diseased donors, acutely infarcted, chronic ischemic, and non-ischemic cardiomyopathy patients. We utilized a panel of 279 antibodies to phenotype cell states that emerge in disease to comprehensively define human cardiac immune-fibroblast diversity and to uncover cell surface protein targets for diagnostic and therapeutic applications. These studies included fresh cells from acutely infarcted hearts, which represents an important milestone towards characterizing the cellular diversity at early stages of heart failure pathogenesis. Moreover, we evaluated mouse cardiac injury models and human fibroblast cell culture systems to assess the suitability of experimental models to recapitulate human cardiac fibroblast phenotypes. Using cell interaction analysis and spatial transcriptomics in human MI tissue, we identified immune-fibroblast neighborhoods within areas of fibrosis that were predicted to be driven by IL-1β signaling from macrophages to activated fibroblasts via a *RUNX1* gene regulatory network. Finally, we demonstrated that IL-1 signaling to fibroblasts is essential for cardiac fibrosis in mice and elucidated that IL-1 signaling coordinates the emergence and maturation of a pro-fibrotic fibroblast trajectory marked by FAP and THY1 expression that resemble matrifibrocytes. These findings establish a causal role for inflammation in fibroblast lineage specification and tissue fibrosis.

## Results

### Multi-omics defines the cellular landscape of human myocardial infarction and heart failure.

**Figure 1.**
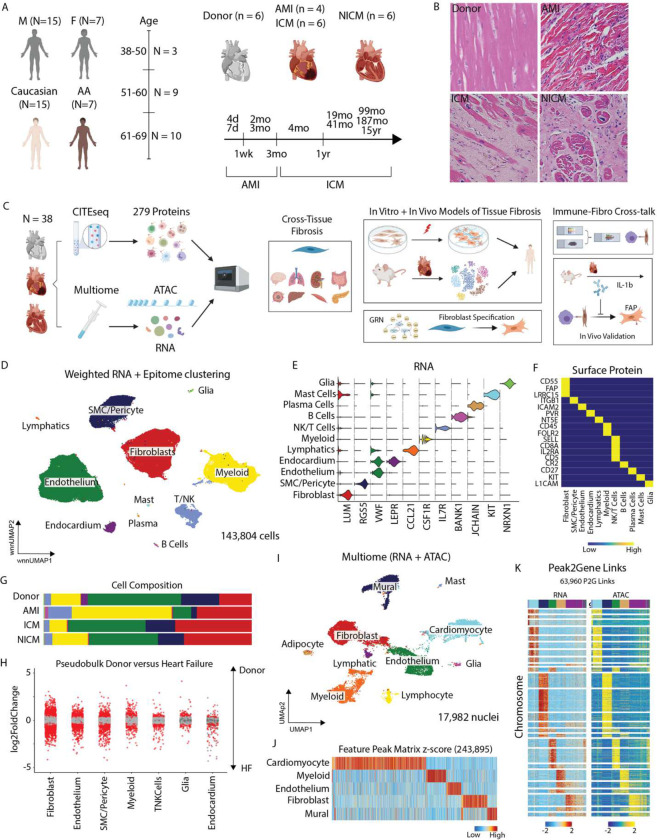
Study design and integrated multi-omic characterization of human MI and HF. (A) Multiethnic diverse patient demographics. (B) Representative H&E sections from donor, acute MI, ischemic cardiomyopathy, and non-ischemic cardiomyopathy. (C) Study design from multi-omic human sequencing derived hypothesis, cross-tissue fibrosis analysis, *in vitro* and *in vivo* experimental characterization, immune-fibroblast crosstalk, and *in vivo* validation studies. (D) UMAP embedding plot with weighted nearest neighbor clustering (RNA and protein) of CITE-seq data from 22 patients and 143,804 cells. (E) Violin plot for canonical marker genes for cell types. (F) Heatmap of marker proteins for cell types. (G) Cell cluster composition across conditions. (H) Pseudobulk differentially expressed genes between donor and HF samples in major cell types (bottom). Red dots indicate statistically significant genes (log2FC > 0.58 and adjusted p-value < 0.05). (I) UMAP embedding plot of RNA from Multiome (paired RNA and ATAC) specimens from 9 patients and 17,982 nuclei. (J) Marker peaks for the major cell types annotated in (I) with FDR < 0.1 and log2FC > 0.5. (K) Peak to Gene linkage heatmap using paired RNA and ATAC information from the same nucleus.

To characterize the transcriptional and surface proteomic landscape of human MI and heart failure, we performed CITE-seq in human left ventricle (LV) specimens obtained from 6 healthy donors, 4 acute MI patients (<3 months post-MI), 6 ischemic cardiomyopathy (ICM, >3 months post-MI) patients, and 6 non-ischemic cardiomyopathy (NICM, idiopathic dilated cardiomyopathy) patients (Fig. 1A). Donor hearts were obtained from brain dead individuals with no known cardiac disease and normal LV function. Acute MI, ICM, and NICM specimens were obtained at the time of left ventricular assist device (LVAD) implantation or heart transplantation. We included samples from males and females (**Table 1**). Hematoxylin and eosin (H&E) staining showed marked inflammation in acute MI specimens and robust fibrosis in ICM and NICM specimens (Fig. 1B).

CITE-seq using 279 surface antibodies was performed on transmural myocardial specimens obtained from the LV anterior wall (Fig. 1C, Supplementary Fig. 1A). After pre-processing and application of quality control filters, we obtained high-quality transcriptomic and proteomic reads in 143,804 cells (Supplementary Fig. 1B–C) and performed data integration and weighted nearest neighbor (WNN) clustering using RNA and protein expression. Differential gene expression (DGE) was subsequently used to identify 11 distinct cell types (Fig. 1D, Supplementary Fig. 1D–F) with canonical gene (Fig. 1E, Supplementary Fig. 2A) and protein (Fig. 1F, Supplementary Fig. 2B) signatures.

We aobserved marked differences in cell type composition across conditions (Fig. 1G, Supplementary Fig. 1F). Increased proportions of myeloid and T-cells and reduced endothelial cell abundance was observed following acute MI with the greatest expansion in myeloid cells occurring within the first week. Accumulation of fibroblasts was seen across pathological conditions beginning 1 week after acute MI. To assess cell specific transcriptional changes in heart failure (HF), we performed pseudobulk DGE analysis at the patient level and observed strong differences between donor and HF (acute MI, ICM, NICM) conditions within fibroblasts, endothelial cells, smooth muscle cells (SMCs) and pericytes, and myeloid cells (Fig. 1H).

To dissect the epigenetic landscape of MI and HF, we performed Multiome (paired single nucleus RNA and ATAC) sequencing. After pre-processing and quality control, we used canonical RNA markers to annotate nuclei (Fig. 1I). Using RNA derived annotations, we called peaks and found feature marker peaks for the major cell types (Fig. 1J). Correspondence between RNA and ATAC-seq modalities was visulaized by constructing a heatmap of the peak-to-gene links split by the two modalities (Fig. 1K).

### Expansion of pathologic fibroblast subsets in acute MI and chronic heart failure

**Figure 2.**
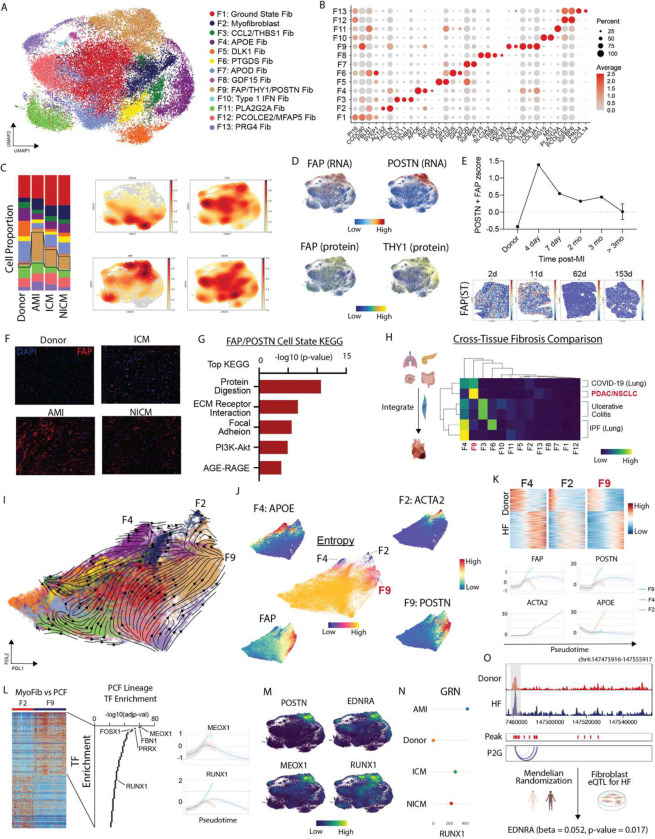
FAP fibroblasts expand post-MI into a PF lineage with a *MEOX1/RUNX1* orchestrated gene regulation network. (A) UMAP embedding of CITE-seq fibroblast cell states. (B) Dot Plot of marker genes for fibroblast cell states. (C) Fibroblast cell state composition across four groups (left) and Gaussian kernel density estimation of cells across four groups (right). (D) FAP RNA (top left), POSTN RNA (top right), FAP protein (bottom left), and THY1 protein (bottom right). (E) FAP/POSTN z-score in donor, acute MI, and ICM split by time from MI with spatial transcriptomics samples showing FAP expression at different time points post-MI in human LV specimens. (F) Immunofluorescence of FAP in donor, acute MI (4 days post-MI), ICM, and NICM left ventricle myocardium. (G) Top KEGG pathways for FAP/POSTN cell state. (H) Cross-tissue fibrosis Pearson correlation coefficient between human heart fibroblast cell states and those from other disease contexts. (I) scvelo analysis in force directed layout embedding and (J) Palantir derived entropy with terminal cell states noted and marker genes for terminal states in addition to FAP plotted in FDL embedding highlighting *FAP* derived fibroblasts diverge into myofibroblats and PFs. (K) Heatmap of pseudobulk differentially expressed genes between donor and HF and terminal state marker genes over pseudotime. (L) Heatmap of differentially expressed genes between F2 and F9 fibroblast cell states (left), TF enrichment analysis for F9 lineage using enrichR (TF-Gene Co-occurrence database) shows key epigenetic regulators of the F9 lineage (middle), and *RUNX1/MEOX1* gene expression across pseudotime for 3 lineages showing increased expression along F9 lineage (right). (M) Gene expression density plot in UMAP embedding for *POSTN*, *RUNX1*, *EDNRA*, and *MEOX1* (markers distinguishing F2 and F9 lineages). (N) CellOracle gene regulatory network betweenness centrality score for *RUNX1* by HF etiology – a higher score indicates that the TF has a greater influence on informational flow in the GRN. (O) ATAC-seq pseudobulk tracts from multiome fibroblasts with peak2gene linkages showing *EDNRA* locus split by donor and HF; Mendelian Randomization shows that there was a significant positive association between genetically predicted *EDNRA* gene expression levels and HF in fibroblasts (beta = 0.052, se = 0.022, and p-value = 0.017).

Unbiased clustering, harmony integration to correct for batch effects, and subsequent DGE identified 13 distinct fibroblast cell states (Fig. 2A) marked by classical fibroblast markers (F1, ground state), *ACTA2/TAGLN* (F2, myofibroblasts), *CCL2/THBS1* (F3), *APOE/AGT* (F4), *DLK1/GPX3* (F5), *PTGDS/GPC3* (F6), *APOD/IGFBP5* (F7), *GDF15/ATF5* (F8), *FAP/POSTN* (F9), *ISG15/MX1* (F10), *PLA2G2A* (F11), *PCOLCE2/MFAP5* (F12), and *PRG4/CXCL14* (F13) (Fig. 2B). Construction of gene set and surface protein signatures for each fibroblast cell state confirmed clear delineation between fibroblast states (Supplementary Fig. 3A–D). Gene Ontology (GO) analysis based on differentially expressed marker genes identified unique pathway enrichment across states (Supplementary Fig. 4A). Spatial transcriptomics data from human heart indicated that fibroblast populations were located within defined perivascular and interstitial spatial niches (Supplementary Fig. 4B–D)^28^.

Fibroblast cell states differed across donor, acute MI, and chronic heart failure conditions. Donor hearts contained the greatest number of F4 (*APOE/AGT*) and F5 (*DLK1/GPX3*) fibroblasts. We used an existing Visium spatial transcriptomics dataset (Methods) from healthy LV tissue and found strongest enrichment of F4, F5, and F6 fibroblast gene signatures – F9 and F13 showed almost no signal while F2 was enriched in the perivascular space (Supplementary Fig. 4B, C). Acute MI and chronic heart failure patients contained the greatest proportion of F9 (*FAP/POSTN*) and F2 (myofibroblasts) (Fig. 2C). F9 fibroblasts were strongly enriched for *FAP* at the RNA and cell surface protein levels co-localizing with *POSTN* (Fig. 2D). Differential expression analysis from the cell surface protein data identified additional markers for the F2 (F11R) and F9 (THY1, CD276, LAMP1, CD63) states (Fig. 2D, Supplementary Fig. 3C–D). In addition to differing transcriptional signatures, pathway enrichment, and protein markers, F2 and F9 fibroblasts resided within distinct regions of the myocardium (Supplementary Fig. 4D). F2 fibroblasts were localized in perivascular regions, while F9 fibroblasts were associated with fibrosis and collagen expression. Collectively, these data highlight that myofibroblasts and FAP/POSTN fibroblasts represent distinct disease associated fibroblast populations. Given the fibrosis associated transcriptional signature of F9 fibroblasts and their localization within the infarct and fibrotic regions of the myocardium, we chose to focus our analysis on this population.

Quantification of the proportion of F9 fibroblasts in individual patients demonstrated consistent increases in acute MI, ICM, and NICM compared to donors (Supplementary Fig. 4E). Temporally, the *FAP/POSTN* transcriptional signature peaked early after MI, declined over a 3-month period, and remained chronically elevated thereafter (Fig. 2E). To validate our findings, we utilized spatial transcriptomics data from human MI at days 2, 11, 62, and 153 post-MI. Within this dataset, *FAP* expression was most prominent acutely after MI and diminished at later time points (Fig. 2E). Immunostaining for FAP in LV sections showed no expression in donors, robust expression following acute MI, and moderate expression in ICM and NICM (Fig. 2F). We next performed pseudobulk DGE analysis between donor and diseased fibroblasts and created gene set signatures for donor and diseased fibroblasts using genes with p-adjusted <0.05, baseMeanExpression >500, and log2FC >0.58. The diseased signature localized specifically within F9 fibroblasts, while the donor signature localized within F5, F7, and F11 fibroblasts (Supplementary Fig. 4F). KEGG pathway analysis using F9 marker genes (p-adjusted <0.05 and log2FC >0.58) revealed enrichment for ECM remodeling, cell-cell adhesion, protein digestion, PI3K-Akt signaling, and AGE-RAGE pathways (Fig. 2G).

To assess how cardiac fibroblasts that emerge in acute MI and chronic heart failure compare to fibroblasts from other disease contexts, we utilized a published single-cell dataset that examined diseased fibroblasts in 3 human tissues: pancreas (cancer), intestine (ulcerative colitis), and lung (COVID-19, non-small cell lung cancer, idiopathic pulmonary fibrosis)^29^. We re-normalized the published data with SCTransform, integrated it with our human cardiac fibroblast dataset, and performed clustering using RNA information (Supplementary Fig. 5A–C). To assess similarity among integrated fibroblast clusters, we calculated Pearson correlation coefficients between pairwise cell states and found that most cardiac fibroblasts differed from fibroblasts found within other tissues and diseases. F9 fibroblasts were most similar to cancer fibroblasts and F4 fibroblasts were similar to IPF fibroblasts (Fig. 2H).

To map fibroblast trajectories in acute MI and heart failure, we undertook two approaches. First, we used scVelo with dynamical modeling to infer directionality in fibroblast cell state differentiation. Projection of the velocity vector field in a force directed layout suggested convergence toward the F2, F4, and F9 fibroblast states (Fig. 2I). Next, we used Palantir to predict fibroblast differentiation endpoints, which also identified F2, F4, and F9 as possible terminal states with low entropy values across a range of simulation parameters (Fig. 2J). To glean how fibroblasts might evolve over the time course of disease, we plotted donor and diseased fibroblast signatures (Supplementary Fig. 4F) versus pseudotime along each predicted lineage (F2, F4, F9). The donor fibroblast signature was most enriched early in pseudotime, while the diseased signature increased over pseudotime in each lineage. *FAP* expression transiently increases along the F2 and F4 lineages and progressively increases along the F9 lineage. Notably, the markers of each population increase over pseudotime in a lineage specific manner (Fig. 2K). To dissect putative regulators of the different trajectories, we identified differentially expressed genes and used the resultant gene list to perform a TF enrichment analysis. This approach identified several transcription factors such as (*FBN1, PPRX,* and *FOSX1*) and the previously characterized *MEOX1*^30^ as the top TF driving the F9 lineage (Fig. 2L). Gene expression density plots showed that *MEOX1* was co-expressed with *RUNX1*, *EDNRA, POSTN* along the F9 lineage (Fig. 2M). Furthermore, *RUNX1* and *MEOX1* increased along the F9 lineage but not the other lineages (Fig. 2L). Using PathwayNet we found that *POSTN*, *RUNX1*, and *EDNRA* are predicted to be connected via a transcriptional regulation network (Supplementary Figure. 4G). Consistent with this finding, multiome fibroblast ATAC pseudobulk tracts revealed increased chromatin accessibility at the *EDNRA* locus in HF relative to donor fibroblasts (Fig. 2N). To explore genetic associations between F9 fibroblasts and HF, we utilized Mendelian Randomization and found an association between genetically predicted *EDNRA* gene expression levels in fibroblasts and HF (beta = 0.052, se = 0.022, and p-value = 0.017) (Fig. 2O).

### In vivo mouse cardiac injury systems recapitulate human cardiac fibroblast diversity.

**Figure 3.**
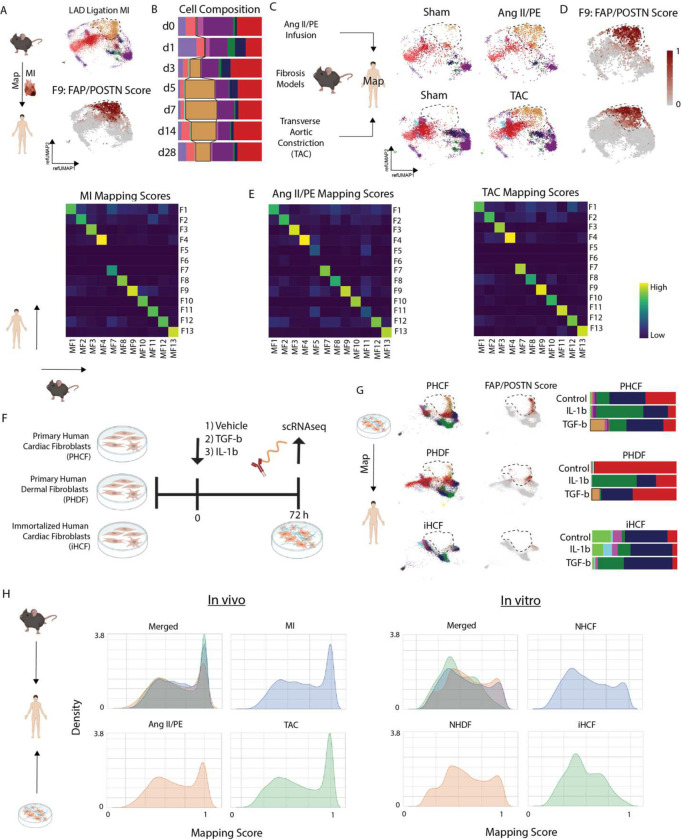
Comparison of *in vivo* and *in vitro* models to study cardiac tissue fibrosis. (A) Reference mapped mouse MI fibroblasts onto human CITE-seq space with label transfer (top) and prediction score for cells annotated in POSTN/FAP cluster. (B) Cell composition from label-transferred cell states post-MI. (C) Reference mapped mouse fibrosis model fibroblasts from Ang II/PE and TAC split by sham and injury. (D) Prediction score for cells annotated in POSTN/FAP cluster from fibrosis models. (E) Heatmap of average cell prediction score of human fibroblast cell states (y-axis) in annotated mouse fibroblasts (x-axis) in MI, Ang II/PE, and TAC. (F) Experimental set-up of *in vitro* model. (G) Reference mapped *in vitro* cell lines post-stimuli (control, TGF-b, and IL-1b) onto human cardiac fibroblasts and corresponding cell composition. (H) Density plot of maximum prediction score for each cell from *in vivo* and *in vitro* systems merged and split by *in vivo* or *in vitro* condition.

To address whether available experimental systems are suitable to study cardiac fibroblasts including the F9 fibroblast, we examined several mouse cardiac injury models and human fibroblast culture systems. First, we sought to address how well a traditional mouse left anterior descending artery (LAD) ligation model mimics human disease using previously published scRNA-seq data obtained from mouse hearts at day 0, 1, 3, 5, 7, 14, and 28 following MI^14^. Raw data was reprocessed using the same computational pipeline employed in our CITE-seq human data to ensure consistency. We first mapped the mouse MI dataset onto the global human CITE-seq reference and detected strong mapping across all populations (Supplementary Fig. 6A–B). Consistent with our human data, we observed increase myeloid infiltration on days 1–7 post-MI (Supplementary Fig. 6C) and phenotypic shifts within fibroblast, myeloid, and endothelial cell populations (Supplementary Fig. 6D–E). We then mapped mouse fibroblasts onto our human CITE-seq fibroblasts using label-transfer to impute cell state annotation. Mapping scores were strong across all most cell states except for F5, F6, and F7 fibroblasts, which were poorly represented in the mouse data (Fig. 3A, Supplementary Fig. 7A). When split by time point, there was marked expansion of F9 fibroblasts that peaked on day 7 post-MI and remained elevated compared to sham on d28 post-MI (Fig. 3B, Supplementary Fig. 7B).

Next, we assessed other mouse cardiac fibrosis models. We infused angiotensin II and phenylephrine (AngII/PE) via implanted osmotic minipump and performed scRNA-seq at day 28 (Supplementary Fig. 8A). After applying QC filters (Supplementary Fig. 8B), data normalization, clustering, and cell type annotation (Supplementary Fig. 8C–D), we selected the fibroblasts and performed label transfer onto our human cardiac fibroblast CITE-seq dataset (Fig. 3C, Supplementary Fig. 8E). Consistent with mouse MI, we recovered most fibroblast cell states except for F5 and F6. We observed expansion of F9 fibroblasts, which were absent in the sham control cohort (Fig. 3C–E). We then examined another published scRNA-seq dataset from hearts that underwent transverse aortic constriction (TAC)^30^. We reprocessed the data and performed label transfer to map mouse TAC fibroblasts onto fibroblasts from our human CITE-seq dataset (Supplementary Fig. 9A–B). Consistent with mouse MI and Ang II/PE infusion models, we found strong mapping scores for most fibroblast populations except for F5 and F6. Robust expansion of F9 fibroblasts relative to sham was also seen (Fig. 3C–E, Supplementary Fig. 9A). Intriguingly, we observed that JQ1 treatment, which resulted in improved LV systolic function and abrogation of fibrosis, was associated with the loss of F9 fibroblasts. Following JQ1 withdrawal, F9 fibroblasts re-emerge suggesting that this is a reversible effect (Supplementary Fig. 9B).

To examine whether commonly used human cultured fibroblast systems model human cardiac fibroblasts, we tested three different fibroblast cell preparations: primary human cardiac fibroblasts (PHCF), primary human dermal fibroblasts (PHDF), and immortalized human cardiac fibroblasts (iHCF). Each fibroblast cell line was treated with vehicle, TGF-β, or IL-1β for 72 hours and scRNA-seq performed. Multiple biological and technical replicates were included, individually hash tagged, and pooled to minimize any potential batch effects (Fig. 3F). After applying QC filters and data normalization, the data was integrated with harmony (Supplementary Fig. 10, 11A–B). To compare human fibroblast preparations to human cardiac fibroblasts, we performed label transfer to map the normalized data onto our human CITE-seq fibroblast dataset and imputed cell annotations. In general, PHCF and PHDF recovered a broader diversity of fibroblast cell states compared to iHCF. However, all cultured cell lines displayed a limited ability to recapitulate fibroblast populations that reside within the human heart. TGF-β treatment led to a modest expansion in F9 fibroblasts in the PHCF and PHDF models, however these cells displayed suboptimal mapping to the human CITE-seq dataset (Fig. 3G). To directly compare the ability of mouse cardiac fibroblasts and human cell culture preparations to recapitulate the diversity of human cardiac fibroblasts, we constructed density histograms of the human CITE-seq mapping scores. Fibroblasts recovered from the mouse cardiac injury models showed superior mapping scores relative to the human cell culture preparations (Fig. 3H, Supplementary Fig. 11C–D). These data indicate that mouse cardiac injury models are a reasonable experimental approach to explore mechanisms involved in cardiac fibrosis including pro-fibrotic F9 fibroblasts.

### Inflammatory signaling within spatially defined cell neighborhoods drives cardiac fibrosis.

**Figure 4.**
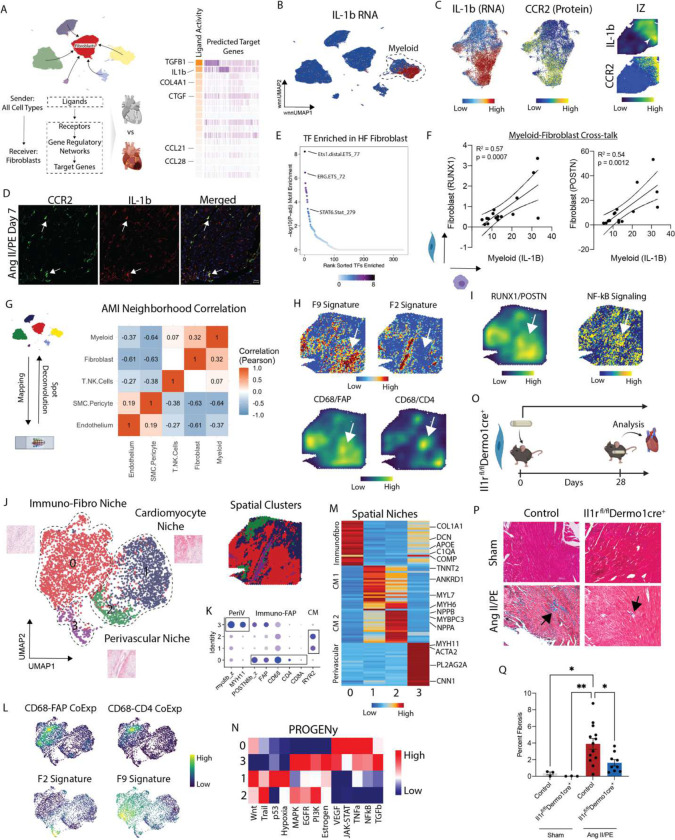
CCR2 macrophages co-localize with and signal to fibroblasts via IL-1b in MI and HF. (A) NicheNet derived predictions of enriched signaling from other cell types to fibroblasts in HF (acute MI, ICM, NICM) relative to donor. (B) IL-1b RNA expression in global CITE-seq UMAP embedding. (C) IL-1b RNA expression in myeloid subsets (left) with corresponding CCR2 protein expression (middle), and IL-1b expression density and imputed CCR2 protein expression in an acute MI IZ spatial transcriptomic sample. (D) Immunofluorescence of IL-1b (red) in CCR2 GFP (green) mice at day 7 post Ang II/PE mini-pump implantation. (E) Rank sorted TF motifs enriched in HF fibroblasts relative to donor fibroblasts from Multiome data show enriched inflammatory signaling. (F) Average expression of IL-1B in macrophages per HF patient versus *RUNX1* (left) and *POSTN* (right) in fibroblasts from the same patient. R^2^ indicates the regression coefficient and the p-value tests whether the slope is significantly non-zero. (G) SPOTlight derived cell-cell proportion correlation in acute MI patient. (H) z-score for marker genes in F9 (*POSTN, COMP, FAP, COL1A1, THBS4, COL3A1*) and F2 (*ACTA2, TAGN*) (top) and CD68/CD4 and CD68/FAP joint density embedding plot (bottom). (I) *RUNX1*/*POSTN* co-localization density plot (left) and PROGENy imputed NF-kB pathway score (right). (J) UMAP embedding plot of spatial clusters with three distinct niches and corresponding spatial location of clusters. (K) Dot plot of marker genes and gene set scores for spatial niches. (L) CD68/CD4, CD68/FAP (top) and F2 and F9 fibroblast gene signature (bottom) joint density embedding plot in UMAP embedding. (M) Heatmap of top differentially expressed genes across spatial niches. (N) PROGENy pathway analysis for spatial clusters in (J). (O) Study design for IL-1 receptor deletion in fibroblasts in mice with Ang II/PE minipump implantation, (P) representative images of trichrome staining, and (Q) quantification of fibrosis at day 28.

To identify cell-cell signaling events that might participate in cardiac fibrosis, we used NicheNet to probe ligand-receptor signaling events enriched in diseased conditions relative to donors. We focused on signals received by fibroblasts from any cell type and identified TGF-β and IL-1β as the strongest predicted signals (Fig. 4A). We chose to focus on IL-1β signaling given the availability of FDA approved therapeutics and potential clinical impact. *IL-1β* was specifically expressed in myeloid cells (Fig. 4B). Within the myeloid cell compartment, *IL-1β* expression was observed in *CCR2*^*+*^ monocytes, macrophages, and classic dendritic cells (DC2s) (Fig. 4C, Supplementary Fig. 12A–F). As an orthogonal approach, we plotted *IL-1β* expression in spatial transcriptomics data obtained from a patient following acute MI (day 2) and observed strong expression in the ischemic zone overlapping with the *CCR2*^*+*^ monocytes, macrophages, and cDC2s (Fig. 4C, Supplementary Fig. 12G, H). IL-1β expression was conserved in mouse CCR2^+^ monocytes and macrophages in the AngII/PE infusion model (Fig. 4D). We then leveraged the multiome ATAC-seq information and found that ETS1 and ERG motifs were enriched in HF relative to donor fibroblasts, consistent with the possibility that macrophage-derived IL-1β may signal to fibroblasts in HF (Fig. 4E). To further infer macrophage-fibroblast crosstalk at the patient level we regressed macrophage *IL-1β* expression with fibroblast *RUNX1* and *POSTN* expression and observed a strong correlation (Fig. 4F).

To determine whether myeloid cells are spatially localized within regions that contain F9 fibroblasts in the diseased human heart, we carried out the following analysis. We performed snRNA-seq in 7 acute MI and ICM LV specimens and integrated the data with our previously published heart failure atlas (consisting of donor and NICM hearts) to generate a comprehensive single nuclei map of the healthy, injured, and failing human heart than can be used to annotate the spatial transcriptomic data (Supplementary Fig. 13A). We identified 13 distinct major cell types as using DGE testing. We then selected representative donor, acute MI, and ICM LV spatial transcriptomic datasets for further analysis. Consistent with our prior analyses, *FAP* expression was increased early after MI within the infarct area and remained elevated in ICM (Supplementary Fig. 13B). To localize cell types within space, we first plotted gene signatures for major cell populations and found consistent overlap with anatomic structures (cardiomyocytes, blood vessels, infarct) identified from the H&E images (Supplementary Fig. 13C). To capture cell types which co-localize with fibroblasts in acute MI, we used SPOTlight to deconvolute each spot into its relative cell composition using our reference. After deconvolution, we calculated cell-cell correlations and found fibroblasts most strongly co-localize with myeloid cells and T-cells forming an immune-fibroblast niche. SMCs/pericytes and endothelial cells co-localize in distinct perivascular regions (Fig. 4G). We applied this approach to multiple donors, acute MI, and ICM patient samples and identified similar niches (Supplementary Fig. 14–15). We then plotted gene signatures for fibroblast states that expanded in disease (F2, F9) and found that F9 fibroblasts co-localized with immune-fibroblast niche (*CD68/FAP/CD4*), while F2 fibroblasts were found within the perivascular niche (Fig. 4H). Given the strong correlation between macrophage IL-1β and fibroblast *POSTN/RUNX1* expression at the patient level, we created a co-localization plot of *POSTN/RUNX1* in AMI and found enriched NF-kB pathway signaling in corresponding regions (Fig. 4I).

To examine transcriptional signatures represented in defined niches, we clustered spatial spots within a UMAP embedding and identified 4 spatial clusters (Fig. 4J). Cluster 0 was strongly enriched for fibroblast and immune cell markers, clusters 2 and 3 expressed cardiomyocyte markers, and cluster 3 was enriched with SMC and pericyte markers (Fig. 4K). Cluster 0 co-localized with regions in space enriched for the F9 gene set score derived from the human CITE-seq dataset (Fig. 4L). Co-localization of *CD68/FAP* and *CD68/CD4* was also present in the immune-fibroblast niche. Myofibroblasts (F2) were not present within this niche and instead localized to perivascular regions (Fig. 4M). We then used PROGENy to understand what pathways are enriched in the spatial clusters and found enrichment for NFkB signaling (downstream mediator of IL-1β) in the immune-fibroblast niche. TGF-β signaling was most represented in the perivascular niche (Fig. 4N). To validate our findings in a broader array of patients, we integrated spatial transcriptomic data across 28 samples in UMAP space from donor, acute MI, and ICM patients. We found high *FAP* and *POSTN* expression in neighborhoods containing expression of macrophage markers (Supplementary Fig. 16A–C). These regions contained the F9 fibroblast signature and were highest in the infarct zone (IZ) followed by the fibrotic zone (FZ) and lowest in donors and the remote zone (RZ) (Supplementary Fig. 16D).

To determine whether IL-1b signaling to fibroblasts is necessary for cardiac fibrosis, we generated mice that lack the IL-1 receptor (IL1R) in fibroblasts (*Il1r*^*flox/flox*^*Dermo1-cre* mice^31^). Control and *Il1r*^*flox/flox*^*Dermo1-cre* mice either underwent sham surgery or received Ang II/PE eluting osmotic minipumps (Fig. 4O). Fibrosis was quantified 4 weeks later by trichrome staining. Ang II/PE infusion resulted in increased interstitial fibrosis in control mice. Consistent with the conclusion that IL-1 signaling to fibroblasts promotes cardiac fibrosis, we observed reduced trichrome staining in AngII/PE treated *Il1r*^*flox/flox*^*Dermo1-cre* mice compared to controls (Fig. 4P–Q).

### T cells expand post-MI and display signatures of activation

Cell composition analysis in the global object showed T cell expansion post-MI and integrated spatial transcriptomic analysis showed co-localization within the immune-fibroblast niche. To further characterize T cell subsets, we leveraged the CITE-seq data and performed WNN clustering (Supplementary Figure. 17A). DE analysis showed strong separation cross cell states (Supplementary Figure. 17B). All T cells expressed *TCRab* (Supplementary Figure. 17C), and we see strong separation of CD4 and CD8 T cells at the protein level (Supplementary Figure. 17C). Markers of T cell activation such as *CCL3, CCL4, IFNG,* and *TNF* were most enriched in AMI (Supplementary Figure. 17D). T cell exhaustion markers such as *CTLA4, PDCD1, HAVCR2*, and *LAG3* were most enriched in the chronic HF patients (Supplementary Figure. 17D). T cell maturation markers such as *CXCR4* and *CD69* were also enriched in ICM and NICM patients relative to AMI (Supplementary Figure. 17D). *TBX21* (necessary for Th1 polarization) was highest in donors while *GATA3* (necessary for Th2 polarization) was highest in AMI and ICM samples (Supplementary Figure. 17D). We also plotted the lymphocyte activation signature (*CCL3, CCL4, IFNG,* and *TNF*) in space in an AMI sample and see expression within the areas positive for *CD68* and *FAP/POSTN* (Supplementary Figure. 17E).

### IL-1β inhibition impedes FAP fibroblast fate specification and maturation

**Figure 5.**
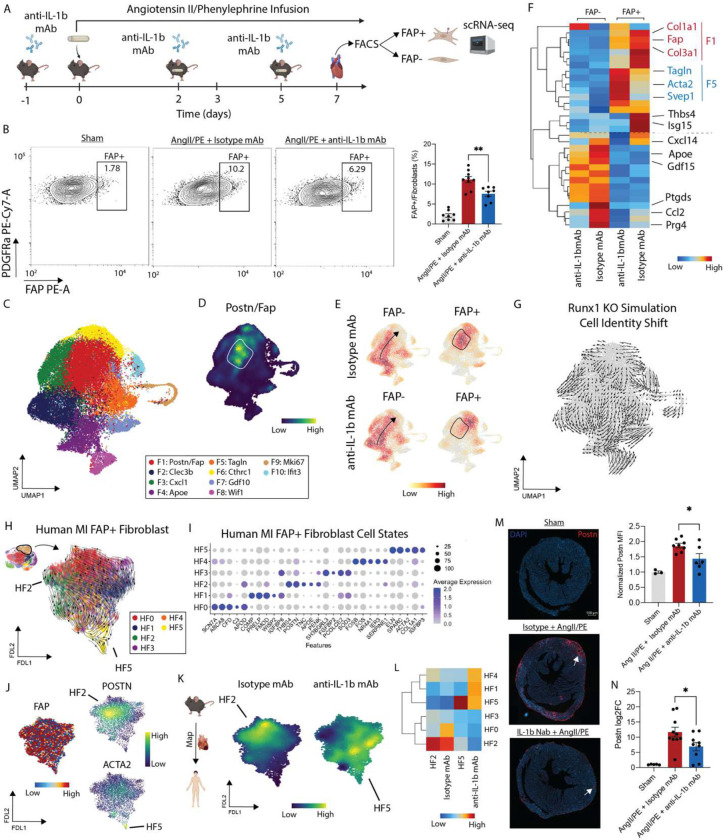
(A) Experimental workflow for cardiac injury experiment with treatment of an IL-1b neutralizing antibody or isotype control. (B) Flow cytometry gating of PDPN vs FAP in a representative sham, Ang II/PE + Isotype, and Ang II/PE + anti-IL-1b mAb mouse heart (left) and FAP+ fibroblasts/total fibroblasts quantified (right). Unpaired t-test and **P = 0.0031 (isotype vs anti-IL-1b mAb). (C) Integrated UMAP for isotype and anti-IL-1b mAb treated mice FAP+/FAP− fibroblasts with annotated clusters. (D) Density plot of Fap/Postn co-localization with area of maximal expression highlighted. (E) Gaussian kernel density plots of 4 conditions in integrated UMAP embedding. (F) Heatmap of key fibroblast cell state genes grouped by four conditions with rows clustered by similarity. (G) CellOracle *Runx1 in silico* knockout simulated cell identify shift shows a shift away from Fap/Postn fibroblasts. (H) scVelo of human FAP+ fibroblasts in a FDL embedding space colored by re-clustered data. (I) Top marker genes for FAP+ human CITE-seq re-clustered data from (F). (J) FAP, POSTN, and ACTA2 expression in human FAP+ fibroblasts in FDL embedding. (K) Mapping mouse differentially expressed signature between FAP+ isotype and anti-IL-1b mAb treated mice. (L) Heatmap of gene set signature for HF2 (*THBS4, POSTN, TNC, APOE,* and *PENK*), isotype treated, HF5 (*ACTA2* and *TAGLN*), and anti-IL-1b mAb treated grouped by FAP+ human fibroblast cell state. (M) IF of *POSTN* in a representative sham, Ang II/PE + Isotype, and Ang II/PE + anti-IL-1b mAb heart (left) with quantification (right). Unpaired t-test and *P = 0.0243. (N) RT-PCR of Postn in mouse hearts split by 3 conditions. Unpaired t-test and *P = 0.0392.

To dissect the therapeutic impact of IL-1β inhibition on fibroblast cell state transitions, we implanted mice with Ang II/PE osmotic minipumps treated animals with an IL-1β neutralizing antibody (anti-IL-1β mAb) or isotype control every 3 days (Fig. 5A). Sham mice served as a negative control. To characterize *FAP* fibroblast fate specification we developed a flow cytometry assay to detect FAP cell surface expression on mouse cardiac fibroblasts (Supplementary Figure 18). Mice were harvested after 7 days on AngII/PE treatment and hearts enzymatically digested for flow cytometry. Quantification of flow cytometry data revealed an increase in the proportion of FAP^+^ fibroblasts in AngII/PE treated animals that received isotype antibodies compared to corresponding controls. Importantly, we observed a reduction in FAP^+^ fibroblasts in AngII/PE treated mice that received anti-IL-1β mAb compared to AngII/PE treated mice that received isotype antibody (Fig. 5B) demonstrating that endogenous IL-1β signaling regulates FAP^+^ fibroblast specification.

To delineate whether IL-1β signaling contributes to FAP^+^ fibroblast maturation, we sorted FAP^+^ and FAP^−^ fibroblasts from the hearts of mice that were treated with AngII/PE for 7 days and received either isotype or anti-IL-1β mAb. Sorted fibroblasts were then subjected to scRNA-seq (Fig. 5A). Following application of QC filters and data normalization (Supplementary Fig. 19A–B), cells were clustered into 10 transcriptionally distinct fibroblast cell states (Fig. 5C, Supplementary Fig. 19B). Density plot of *Fap/Postn* co-expression showed strong enrichment in clusters F1 and F6 (Fig. 5D). GO Biological Pathway analysis identified enrichment for fibrosis associated pathways including ECM organization, integrin signaling, and collagen fibrin organization in mouse FAP^+^ fibroblast states (Supplementary Fig. 19C).

To unravel phenotypic shifts among fibroblasts following anti-IL-1β mAb treatment, we constructed Gaussian kernel density embedding plots. As anticipated, significantly different cell distributions were found in the FAP^+^ and FAP^−^ groups with enrichment of cells in the F1 and F6 clusters in the FAP^+^ fibroblast group. Mice that received anti-IL-1β mAb displayed reductions in FAP^+^ fibroblasts that localized to cluster F1 compared to corresponding isotype control (Fig. 5E). We detected differences in gene expression in both the FAP^+^ and FAP^−^ fibroblasts following anti-IL-1β mAb treatment (Supplementary Fig. 19D). Within the FAP^+^ population, isotype treated animals displayed increased expression of *Fap, Thbs4, Isg15, Col1a1, and Cola3.* Conversely, IL-1β mAb treatment increased the expression of myofibroblast markers including (*Tagln*, *Acta2*, and *Svep1*) in FAP^+^ fibroblasts (Fig. 5F). These data suggest that IL-1β mAb treatment may impact the differentiation trajectory of FAP^+^ fibroblasts. GO biological pathway analysis further suggested that anti-IL-1β mAb treatment impacted genes involved in ECM organization, T cell migration, cytokine signaling, and chemokine production (Supplementary Fig. 20D). FAP^−^ fibroblasts showed reduced expression of *ApoE*, *Gdf15*, *Ptgds*, *Ccl2*, and *Prg4* following IL-1β mAb treatment (Fig. 5F). To unravel how *Runx1* impacts fibroblast lineage specification, we used CellOracle to construct cell state specific gene regulatory networks and then performed a *in silico Runx1* perturbation and the simulated cell identity shifts showed movement away from the F1 state (*Postn, Fap*) (Fig. 5G).

To examine the differentiation trajectory of FAP^+^ fibroblasts, we sub-clustered human FAP^+^ fibroblasts (F9 cell state) from our human CITE-seq dataset at finer resolution. DEG analysis showed distinct cell states within the FAP^+^ populations (Fig. 5H–I). All cells expressing *FAP* and only a subset expressing *POSTN* or *ACTA2* (Fig. 5J). To predict differentiated cell states, we used scVelo and observed convergence within the *POSTN* fibroblast (HF2) and *ACTA2* myofibroblast (HF5) clusters (Fig. 5H). Next, we mapped the mouse FAP^+^ fibroblasts onto the human FAP^+^ fibroblast FDL trajectory and plotted gene signatures enriched in following either isotype or anti-IL-1β mAb treatment. We observed a strong separation triggered by anti-IL-1β mAb treatment (Fig. 5K). To interpret the effect of anti-IL-1b mAb treatment within the context of the human FAP^+^ fibroblast differentiation, we created a heatmap of isotype enriched, anti-IL-1b mAb enriched, and human *POSTN* (HF2) signatures grouped by human FAP^+^ fibroblast state. We found that the isotype signature corresponds with the human HF2 (*POSTN*) signature, while the anti-IL-1b mAb signature is enriched for alternative human fibroblasts states including myofibroblasts (HF1, HF4, HF5) (Fig. 5L). These data further suggest that IL-1b mAb treatment impedes the maturation of FAP^+^ fibroblasts into *POSTN* fibroblasts.

To experimentally validate these findings, we performed immunostaining for POSTN in sham hearts and hearts treated with AngII/PE for 7 days. Compared to sham controls, AngII/PE infusion increased the abundance of POSTN^+^ cells. Consistent with our scRNA-seq data, we observed a reduction in POSTN^+^ cells in AngII/PE treated hearts from mice that received anti-IL-1β mAb compared to isotype control (Fig. 5M). Similarly, quantitative PCR revealed upregulation of *Postn* expression in myocardial tissue obtained from AngII/PE treated mice compared to sham controls. Treatment with anti-IL-1β mAb reduced *Postn* expression induced by AngII/PE infusion within the heart relative to isotype control (Fig. 5N).

## Discussion

The advent of single cell multi-omic technologies has afforded the opportunity for the high-resolution detection of novel cell states from healthy and diseased human tissue including the heart^22,23,25,26,28,32^. These human cell atlases have provided new insights into the cellular landscape of HF and identified unexpected cell diversity within the stromal compartment. However, the functional roles of these novel cell states and how they might interact during disease pathogenesis remains to be defined. Among stromal cell types, fibroblasts are of particular interest given their role in tissue fibrosis, arrhythmias, and heart failure^19,33,34^. Little is known regarding the mechanisms and signaling events that orchestrate the specification of pathogenic fibroblasts in the context of human MI and HF. Herein, we utilize CITE-seq and spatial transcriptomics to dissect fibroblast cell states that emerge following acute MI and in chronic HF and identify a fibroblast trajectory marked by *FAP* and *POSTN* expression that are governed by inflammatory cytokines and contribute to cardiac fibrosis.

Unbiased clustering revealed an expansion of fibroblasts expressing *FAP* that peaked early after MI and remained elevated in chronic HF. We used IF and spatial transcriptomics in acute MI hearts^28^ to validate expression of FAP at the RNA and protein levels. Recent studies have utilized chimeric antigen receptor (CAR) T cells to target FAP^+^ fibroblasts in a mouse model of cardiac injury and shown improved cardiac function and diminished fibrosis^9,10^. This points to a potential pathogenic role of FAP^+^ fibroblasts in adverse LV remodeling. Transcriptionally, cardiac FAP^+^ fibroblasts resemble cancer associated fibroblasts, which also express *FAP*^*35*29^. Computational lineage analysis predicted that *FAP* expression marks a fibroblast trajectory that transitions into populations including POSTN^+^ matrifibrocytes^11^ and myofibroblasts. Unraveling the signals that drive the differentiation and maturation of FAP+ fibroblasts may identify new therapeutic targets to limit fibrosis in the injured and failing heart.

A fundamental question that continues to challenge the field is selection of appropriate model systems to investigate human cardiac fibroblasts. To address this issue, we leveraged publicly available and new scRNA-seq datasets to examine the suitability of mouse cardiac injury models (MI, Ang II/PE infusion, TAC) and human cultured fibroblast platforms. Surprisingly, we found that each of the mouse models contained many of the fibroblast populations found in the human heart and recapitulated expansion of *Fap* and *Postn* expressing fibroblasts following injury. In contrast, primary human cardiac fibroblasts, primary human dermal fibroblasts, and immortalized human cardiac fibroblasts were relatively poor models of human cardiac fibroblast diversity under resting or stimulated (TGF-β^23,36,37^, IL-1β^38^) conditions. These findings emphasize the relevance of *in vivo* mouse models in studying cardiac fibrosis and for therapeutic discovery.

Little is understood regarding the signaling mechanisms that underlie the specification and differentiation of pathological fibroblasts in the heart. Using cell-cell interaction analysis, we identified enriched for IL-1β and TGF-β signaling in fibroblasts in HF relative to donor control conditions. While numerous studies have implicated a role for TGF-β signaling in maladaptive cardiac remodeling and scar formation in HF^23,36^, the potential for inflammatory signals to drive fibroblast activation and cardiac fibrosis is less explored. In support of this concept, a large clinical trial targeting the IL-1β pathway with the neutralizing antibody, canakinumab, led to a reduction in ischemic cardiac events^39^. We show that IL-1β is selectively expressed by CCR2^+^ monocytes and macrophages in the human heart consistent with our prior mouse studies^40,41^. Using spot deconvolution and Pearson correlation analysis in spatial transcriptomic data from human hearts, we identified spatial niches of macrophages and T-cells that strongly co-localize with fibroblasts in acute MI. This niche robustly expressed *FAP*, extracellular matrix components associated with fibrosis, and displayed an inflammatory signature. We validated the existence of this immune-fibroblast neighborhood in a larger dataset of 28 patients and observed strong macrophage-fibroblast co-localization in pro-inflammatory niches. Together, these findings suggest that FAP^+^ fibroblasts reside within a spatially defined cell neighborhood that contains inflammatory macrophages and that these cell types may communicate via IL-β signaling.

To verify the importance of IL-1 signaling, we generated mice that lack the IL-1 receptor in fibroblasts and demonstrated a specific requirement for IL-1 signaling to fibroblasts in cardiac fibrosis. Next, we sought to explore the mechanistic implications of inhibiting IL-1β signaling as a therapeutic. We found that anti-IL-1 β mAb treatment abrogated the emergence of FAP^+^ fibroblasts. scRNA-seq of cardiac fibroblasts in this model uncovered an unexpected effect on the maturation of the *Fap* fibroblast trajectory. We observed that FAP^+^ fibroblasts differentiated into POSTN^+^ fibroblasts in isotype control hearts. Strikingly, mice treated with the anti-IL-1 β mAb displayed a differentiation trajectory away from POSTN^+^ fibroblasts and towards the myofibroblast lineage. These findings were validated by immunostaining and highlight a central role of IL-1β signaling in pathogenic fibroblast fate specification. They also suggest that IL-1β targeted therapies may not interfere with structural stability of the heart as myofibroblasts are preserved in this context.

Our study is not without limitations. First, we are not powered to assess the effects of demographics (age, sex, ethnicity) on the cell state diversity post-MI. We included patients of diverse ethnicities, sex, and broad age ranges to remain inclusive in the generation of the human dataset. Second, we have a limited cohort size in the AMI group. Given the challenge acquiring fresh acutely infarcted cardiac tissue, we validated our CITE-seq findings in a broad array of patients through multiple assays such as IF and spatial transcriptomics.

In conclusion, we utilize multi-omic single cell sequencing to characterize immune-fibroblast cross talk in human myocardial infarction and HF. We identify a macrophage subset in humans which signals to fibroblasts via IL-1 β and modulates fibroblast activation, lineage specification via a *RUNX1* orchestrated gene regulatory network, and cardiac fibrosis. We show *in vivo* IL-1 β signaling in fibroblasts drives activation, specification towards PFs, and is causally linked to fibrosis. Collectively, our findings provide the first single cell characterization of immune fibroblast crosstalk in the human heart, particularly in the early stages of a heart attack and highlight a promising role for immunomodulators in targeting cardiac fibrosis. These findings have broad implications for future therapeutic discovery and the emerging field of cardio-immunology.

## Materials and Methods

### Ethical Approval for Human Specimens

The study is compliant with all relevant ethical regulations and has been approved by the Washington University School of Medicine Institutional Review Board (IRB #201104172). Informed consent was obtained from each patient prior to tissue collection by Washington University School of Medicine and no compensation was provided in exchange for subject participation in the study. All demographic and clinical data has been de-identified and provided in Supplementary Table 1. Patients included in this study span diverse race, age, and sex to provide an inclusive trans-ethic study population.

### Inclusion Criteria

Prior to tissue collection, specific inclusion criteria were employed to ensure well controlled study groups. Any patients with HIV or hepatitis and known genetic cardiomyopathies were excluded from this study. Donor hearts: patients with stable ejection fractions, no know history of cardiac disease and a non-cardiac cause of death/transplant. Acute MI hearts: patients who had a myocardial infarction from coronary artery disease within 3 months from the time of transplant. Ischemic cardiomyopathy: patients who had a myocardial infarction from coronary artery disease greater than 3 months from the time of transplant. Non-ischemic cardiomyopathy: patients with HF independent of ischemic heart disease and no known history of a myocardial infarction.

### Human single cell isolation for CITE-seq

Fresh cardiac left ventricular tissue from ex-planted hearts at the time of transplantation, LVAD cores, or donors declared DCD at Washington University School of Medicine and Mid America Transplant Service were harvested, perfused with cardioplegia, and transported on ice. In donor, ICM, and NICM hearts a section of the LV apex was dissected out; in acute MI hearts a section of the infarct region (identified as a white discoloration) was dissected. Tissues were minced using a razor blade on ice and transferred to a 15 mL conical tube containing 3mL DMEM with 170uL Collagenase IV (250U/mL final concentration), 35uL DNAse1 (60U/mL), and 75uL Hyaluronidase (60U/mL) and incubated at 37 °C for 45 min with agitation. After 45 min, the digestion reaction was quenched with 6 mL of HBB buffer (2% FBS and 0.2% BSA in HBSS), filtered through 40 𝜇m filters into a 50 mL conical tube and transferred back into a 15 mL conical tube to obtain tighter pellets. Samples were then spun down at 4 °C, 1200rpm for 5 min and the supernatant was discarded. Pellet resuspended in 1 mL ACK Lysis buffer (Gibco A10492-01) and incubated at room temperature for 5 min followed by the addition of 9 mL DMEM and centrifugation (4 °C, 5 min, 1200 rpm). Supernatant was discarded and the pellet was resuspended in 2 mL FACS buffer (2% FBS and 2mM EDTA in calcium/magnesium free PBS) and centrifugation was repeated in above conditions and supernatant aspirated. The TotalSeq A 277 panel (BioLegend) antibody cocktail was resuspended in 100 uL of FACS buffer and 1 uL each of custom oligo-labeled FAP (Amgen) and LRRC15 (Amgen) were added. The combined 102 uL were used to resuspend the pellet with the addition of 1 uL of DRAQ5 (Thermo Fisher Scientific, 564907) and incubated on ice for 30 min. Solution was washed 3x with FACS buffer following same centrifugation as above and then resuspended in 500 uL of FACS buffer and 1 uL DAPI (BD Biosciences, 564907) and filtered into filter-top FACS tubes. First singlets were gated and subsequent DRAQ5+/DAPI− events were collected in 300 uL cell resuspension buffer (0.04% BSA in PBS) – collected cells were centrifuged as above and resuspended in collection buffer to a target concentration of 1,000 cells/uL. Cells were counted on a hemocytometer before proceeding with the 10x protocol.

### CITE-seq Library Preparation

Collected cells were processed using the using the single Cell 3’ Kit v 3.1 (10x Genomics PN: 1000268). 10,000 cells were loaded onto ChipG (PN:1000121) for GEM generation. Reverse transcription, barcoding, and complementary DNA amplification of the RNA and ADT tags were performed as recommended in the 3’ v3.1 chromium protocol. Single-cell libraries were prepared using the single Cell 3’ Kit v 3.1 following a modified 3’ v3.1 assay protocol (User Guide CG000206) to concurrently prepare gene expression and TotalSeq A antibody derived tag (ADT) libraries as recommended by BioLegend. 1 ul of 0.2uM ADT Additive Primer (CCTTGGCACCCGAGAATT*C*C) and 15 ul of cDNA Primers (PN: 2000089) were used to amplify cDNA. ADT libraries were amplified with a final concentration of 0.25 uM SI Primer (AATGATACGGCGACCACCGAGATCTACACTCTTTCCCTACACGACGC*T*C) and 0.25 uM TrueSeq Small RNA RPI primer (CAAGCAGAAGACGGCATACGAGAT[6nt index]GTGACTGGAGTTCCTTGGCACCCGAGAATTC*C*A) using 15 cycles. Gene expression libraries were indexed using Single Index Kit T Set A (PN: 2000240). Libraries were sequenced on a NovaSeq 6000 S4 flow cell (Illumina).

### CITE-seq alignment, quality control, and cell type annotation

Raw fastq files were aligned to the human GRCh38 reference genome (v) using CellRanger (10x Genomics, v6.1) with the antibody capture tag for the TotalSeqA 277 + two custom oligo-tagged antibodies. First, protein reads were normalized and de-noised for each sample separately using the dsb package^42^ in R v4 with the isotype controls tag to remove noisy cell-to-cell variation. Subsequent quality control, normalization, dimensional reduction, and clustering was performed in Seurat v4.0. Following normalization, quality control was performed and cells passing the following criteria were kept for downstream processing: 500 < nFeature_RNA < 600 and 1,000 < nCount_RNA < 25,000 and percentage mitochondrial reads < 15%. Raw RNA counts were normalized and scaled using SCTransform^43^ regressing out percent mitochondrial reads and nCount_RNA. Principal component analysis was performed on normalized RNA counts and to determine the number of PCs to use for further processing the following criteria was applied: PCs exhibit cumulative percent > 90% and the percent variation associated with the PCs as < 5%. Weighted nearest neighbor clustering^44^ (WNN) was performed with the significant RNA PCs and dsb normalized proteins (minus isotype controls) directly without PCA as previously outlined with the FindMultiModalNeighbors function in Seurat. Subsequently, a uniform manifold approximation (UMAP) embedding was constructed and FindClusters was used to un-biasedly cluster cells using the SLM modularity optimization algorithm. Clustering was performed for a suite of different resolutions (0.1–0.8 at 0.1 intervals) and differential gene expression using the FindAllMarkers function and a Wilcoxon Rank Sum test with a logFC cut-off of 0.25 and a min.pct cut-off of 0.1. Clusters were annotated using canonical gene and protein markers and subsequent violin plots (RNA) and heatmaps (protein) were created to assess clean separation of clusters into distinct cell types. Previously identified canonical marker genes were also plotted on the UMAP object to further validate cluster annotations. Cell composition in donor, acute MI, ICM, and NICM was calculated as percentages as previously described. Cellular density profiles in the WNN UMAP space were calculated in Scanpy per condition using the tl.embedding_density function. Briefly, a Gaussian density kernel estimation is used to estimate cell density in WNN UMAP space in each condition (donor, acute MI, ICM, and NICM) and density values are scaled between 0–1. Similar approach used for all cell density calculations in manuscript.

### Cell composition and density shift calculations

We used R to compute cell type composition across conditions. To assess shifts in cell density within both the global object and within individual cell types, we converted the .rds object to a .h5ad file format and used scanpy.tl.embedding function which employs a Gaussian kernel density estimation of cell number within the UMAP embedding. Density values are scaled from 0–1 within that category.

### Pseudobulk Differential Gene Expression

We use the DESeq2^45^ package to perform pseudobulk DE analysis. After QC, cells were subsetted for each cell population annotated from the global reference, raw counts were aggregated to the patient level, data normalized using a regularized log transform, and differential expression analysis between donor and all HF (acute MI, ICM, NICM) via DESeq2. Genes were deemed statistically significant if adjusted p-value < 0.05 and absolute(log2FC) > 0.58. For constructing pseudobulk donor/HF gene set scores, we only used statistically significant genes with a mean log base expression of > 500.

### Multiome sample preparation

Nuclei were isolated according to 10x Genomics protocol (CG00375; Nuclei Isolation Complex Sample for ATAC GEX Sequencing RevB) and flow cytometry for 7-AAD (Sigma; SML1633-1ML) positive nuclei was used for sorting. Protocol CG000338 from 10x Genomics was used for Chromium Next GEM Single Cell Multiome ATAC + Gene Expression. Briefly, following nuclei isolation, permeabilization was performed, followed by transposition, GEM generation and barcoding using ChipJ (10x Genomics; PN1000234), post-GEM clean up, pre-amplification PCR, cDNA amplification, library construction, and sequencing. Gene expression and ATAC libraires were sequenced to a read depth of 50,000 and 25,000 respectively.

### Multiome data processing

Raw fastq files were aligned to the human GRCh38 reference genome (v) using CellRanger ARC (10x Genomics, v6.1). ArchR (https://www.archrproject.com) was used to process the ATAC fragments and Seurat was used to process RNA. Quality control was performed to keep nuclei with the following: TSS enrichment > 2, nFrags > 1000, 200 < nUMI GEX < 50,000, and percent mito < 5%. Post-QC nuclei were used for doublet removal in ArchR (ATAC information) and then using scrublet (RNA information). Raw RNA counts were normalized and scaled using SCTransform^43^ regressing out percent mitochondrial reads and nCount_RNA. Principal component analysis, harmony batch integration (by sample), nearest neighbor clustering, and UMAP embedding construction was then performed in Seurat. Cell types were annotated using different expression and knowledge of canonical gene markers. The RNA annotations and normalized gene expression matrix was added to the ArchR project onto the nuclei with the same barcodes. Using these annotations, pseudobulk replicates were constructed in ArchR followed by peak calling with MACS2. Marker peaks were then defined for the major cell types and a differential accessibility analysis was run between donor and HF samples for motif enrichment. chromVAR was used to perform peak2gene linkage using the ground truth gene expression matrix. Pseudobulk tracts were created in ArchR.

### Fibroblast, myeloid, and T cell states analysis

To cluster cell types into distinct cell states, we subsetted the cell type of interest, re-normalized, computed PCAs, harmony integrated, computed UMAPs, and clustered data at a range of resolutions. DE analysis was then used to identify marker genes for each cell state and a dot-plot or heatmap to assess clustering separation. Using the top marker genes we calculated gene set z-scores and plotted them in UMAP space.

### Perturbed Disease Associated Fibroblasts

Perturbated state human fibroblasts were used from (E-MTAB-10324)^29^ processed using the same pipeline as used for human CITE-seq fibroblasts. Seurat was then used to integrate the perturbed state human diseased fibroblasts with human HF CITE-seq fibroblasts. To assess cluster similarity, we used two approaches: (1) built a phylogeny tree using the BuildClusterTree function and (2) computed Pearson correlation coefficients and displayed results as a matrix.

### Pseudotime Trajectory Analysis

Palantir: Palantir was used to perform pseudotime analysis on fibroblasts and macrophage subsets. For the cell type of interest, a normalized count matrix was exported and processed in python via Palantir. A force directed layout was then computed for visualization of trajectories and MAGIC was used to impute data for visualization. For fibroblasts, F1 was used as the starting cell state and for myeloid cells monocytes were used as the starting cells and resident macrophages were excluded from the trajectory analysis. No terminal states were specified. Generalized Additive Models within Palantir was then used to compute gene expression trends along the different fibroblast lineages. Pseudotime, entropy, and FDL embedding values were exported and subsequent plotting for visualization was performed in R/Seurat. scVelo: Using CellRanger aligned BAM files as input, a loom file containing the estimation of spliced and unspliced RNA counts were generated by Velocyto CLI (v0.17.17)^46^. The loom file was further merged with annotated fibroblasts object and pre-processed, RNA velocity was estimated using dynamical or stochastic modeling and the fdl embedding stream graph was generated by scvelo (https://github.com/theislab/scvelo).

### Pathway Analysis and transcription factor enrichment

Statistically significant DE genes were used to perform pathway analysis via EnrichR (https://maayanlab.cloud/Enrichr/). Pathway enrichment values were downloaded as .csv files and plots generated in Prism. GO enrichment analysis to compare cell states was done in R using clusterProlifer^47^ compareClusters function using only statistically significant marker genes. PROGENy^48^ was used for pathway analysis in the spatial transcriptomics dataset.

### In Vitro Fibroblast single-cell experiments

Human ventricular cardiac fibroblasts (NHCF-V, Lonza, CC-2904), adult human dermal fibroblasts (NHDF-Ad, Lonza, CC-2511), and immortalized human cardiac fibroblasts (iHCF, Applied Biological Materials Inc., T0446) were grown in FibroGRO LS medium (Millipore, SCMF002) supplemented with 2% (NHDF-Ad) or 10% (NHCF-V and iHCF) FBS. Cells were maintained in a humidified atmosphere (95% air, 5% CO_2_) at 37°C and passaged every 2–3 days. For cytokine treatment, cells were seeded in 6-well plates and allowed to attach for 24hr. Thereafter, cells were equilibrated for 16 hr in low serum medium (0.2% FBS for NHDF-Ad, and 0.5% for NHCF-V and iHCF), and then treated with 10 ng/mL of recombinant human TGF-b (R&D Systems; 240-B-002) or 10 ng/mL of recombinant human IL-1β (R&D Systems; 201-LB-005) for the indicated times. After trypsinization, cell suspensions were labeled with barcoded lipids from the 3' CellPlex Kit Set A (10x Genomics PN:1000261) following manufacturer’s instructions (Protocol CG000391). After labeling, technical replicates were pooled and loaded onto a Chromium Controller (10x Genomics) at a concentration to capture 6–10,000 cells. Single-cell libraries were prepared using the Single Cell 3’ Kit v3.1 (10x Genomics PN: 1000268) according to manufacturer’s instructions (User Guide CG000388). Gene expression and cellplex libraries were indexed with Dual Index Kit TT Set A and NN Set A indexes (PN: 1000215, 3000482) respectively. Libraries were pooled and sequenced on a NovaSeq 6000 (Illumina).

### Processing of in vitro single cell data

Raw fastq files were aligned to the human GRCh38 reference genome using CellRanger (10x Genomics, v6.1). In vitro single-cell data was processed using the same pipeline as human CITE-seq data for iHCF, NHDF, and NHCF datasets.

### Reference mapping of in vivo and in vitro data

In vitro: The same mapping pipeline was used for iHCF, NHDF, and NHCF datasets. Briefly, SCTransform normalized data was used, the FindTransferAnchors function was used with a spca reference reduction and 50 components. The anchors were then passed into the MapQuery function to impure cell annotations and project the in vitro data into the human CITE-seq fibroblast umap embedding. Mapping scores for each imputed cell state were then plotted in the umap space and a heatmap of average mapping score for each cell state was constructed.

In vivo: for the day 28 Ang II/PE, TAC, and MI the mouse genes were first converted into the human analogs using biomaRt, the data was subsequently normalized using SCTransform, and normalized data was used to find anchors and then projected onto the human CITE-seq fibroblast UMAP space as described with the in vitro data. Mapping of in vivo and in vitro used the same pipeline and parameters.

### Human single nuclei isolation for snRNA-seq

Cardiac tissues from LVAD cores at the time of LVAD implant (U/RR-pre) and adjacent to core samples at the time of explant (U/RR-post) from paired patient were flash frozen using liquid nitrogen. Identical regions from the apex of LV from explanted donors were used. Single nuclei suspensions were generated as previously described. In brief, flash frozen sections were minced with a razor blade, transferred to a Dounce Homogenizer containing 1 mL of lysis buffer (10 mM Tris-HCl, pH 7.4, 10 mM NaCl, 3 mM MgCl_2_ and 0.1% NP-40 in nuclease-free water) on ice. Samples were homogenized using five strokes, an additional 1 mL of lysis buffer added, and incubated on ice for 15 min. Samples were then filtered with a 40 um filter, which was rinsed with 1 mL of lysis buffer. The mixture was then centrifuged at 500 *g* for 5 min 4 °C, resuspended in 1 mL nuclei wash buffer (2% BSA and 0.2 U ul^−1^ RNase inhibitor (Thermo Fisher, cat. no. AM2694) in 1X PBS) and, filtered using a 20 um pluristrainer (Pluriselect, cat. No. SKU43-50020-03). Filtered solution as centrifuged using the above criteria and resuspended in 300 uL Nuclei Wash Buffer and transferred into a 5 mL tube for flow cytometry. Subsequently, 1 uL DRAQ5 (5 mM solution; Thermo Fisher, cat. no. 62251) was added, sample gently vortexed and allowed to incubate for 5 min prior to sorting. DRAQ5^+^ nuclei were sorted into 300 uL Nuclei Wash Buffer using a BD FACS Melody (BD Biosciences) with a 100 uM nozzle. Sorted nuclei were then centrifuged using the above conditions and resuspended in Nuclei Wash Buffer for a final target concentration of 1,000 nuclei/uL – nuclei were counted on a hemocytometer. Based on the nuclei concentration, 10,000 target nuclei were loaded onto a Chip G for GEM generation using the Chromium Single Cell 5ʹ Reagent v1.1 kit from 10X Genomics. Reverse transcription, barcoding, complementary DNA amplification and purification for library preparation were performed as per the Chromium 5ʹ v1.1 protocol at the McDonnel Genome Institute. Sequencing was performed on a NovaSeq 6000 platform (Illumina) at a target read depth of 100,000 at the McDonnel Genome Institute.

### snRNA-seq reference map generation

Nuclei fastq files were aligned to the whole genome pre-mRNA reference generated from the GRCh38 transcriptome (with the intron flag included) using CellRanger v3 (10X Genomics). Nuclei were filtered to include those with 1000 < RNA UMI count < 10,000 and mitochondrial reads < 5%. After initial QC, scrublet was ran on each sample separately in Python with default parameters to score nuclei and nuclei with a doublet score > 0.2 were excluded from downstream analysis. We then leveraged supervised doublet removal as previously described on a per cell type basis before combining all objects. Briefly, clusters were annotated into major cell populations, each major cell type was subsetted, and re-normalized, PCA, UMAP embedding, clustering, and DE analysis performed. Sub-clusters which did not express the gene signature of the cell type or had overlapping genes across different cell types were removed. Post-contamination, all cell type objects were merged to construct a cleaned object. After QC and doublet removal downstream analysis was performed in Seurat v4^49^. The cleaned object was normalized using SCTransform^43^ with regressing out mitochondrial percent and RNA UMI counts. We then computed the principal components and used these to integrate all samples with harmony^50^. Informed by the ElbowPlot, we used 80 components to construct the UMAP embedding, find nearest neighbors, and clustered the data at multiple resolutions. We then used the FindAllMarkers function in Seurat to perform differential expression testing and annotated clusters into distinct cell types based on canonical gene markers. To define cell states within each major cell type, we subsetted the major cell populations, re-normalized, re-clustered, re-computed the UMAP, and annotated cell states. Both the global object and each cell type object was saved and used for downstream analysis and plotting in Seurat. All differential gene expression used to identify cell types or cell states was performed using the normalized assay with the FindAllMarkers function and the Wilcoxon rank sum test with a min.pct = 0.1 and logfc.threshold = 0.25.

### Integration of spatial transcriptomics data

Visium 10x data from 10x Genomics was used in Supplementary Figure 3 (https://www.10xgenomics.com/resources/datasets/human-heart-1-standard-1-1-0) – processed as per Seurat v4. Processed .h5ad objects were acquired for all spatial transcriptomic samples as previously published^51^. Data was re-normalized using SCTransform to keep analysis consistent with the human data processing. The snRNA-seq reference map was used to impute voxel annotations from selected spatial transcriptomic samples – mapping scores and gene signatures were also plotted to validate mapping. SPOTlight^52^ was used for spot deconvolution and Pearson correlation coefficients calculated to identify cells which co-localize in space. To validate findings from isolated patients in a broader array of patients, the above analysis was performed on several samples and an integrated UMAP embedding of spatial spots was constructed for 28 samples using the same analysis pipeline as for snRNA-seq reference map construction.

### Receptor-ligand interaction analysis

Using fibroblasts as a receiver, we first used nichenetr (v1.1.0) default pipeline to explore the top 20 ligands from all other non-fibroblast cell types, that are predicted to orchestrate the highest regulatory potential to regulate downstream target expression in fibroblasts. The regulatory potential from top 20 ligands on their predicted targets in fibroblasts was represented in a heatmap. To further capture the disease altered signaling from myeloid cells to fibroblasts, we extended the analysis to draw information from the differential expression of the ligand-receptor pairs across HF groups (HF vs healthy) by using the differential NicheNet pipeline. Circos plot was created to visualize the interaction between ligands from myeloid cells and receptors from fibroblast cells.

### Mouse strains

All animal studies were performed in compliance with guidelines set forth by the National Institutes of Health Office of Laboratory Animal Welfare and approved by the Washington University institutional animal care and use committee. All mouse strains used in this study (WT, CCR2 GFP) are on the C57BL/6 background. Il1r^fl/fl^ mice were crossed to the Dermo1 cre to generate Il1r^fl/fl^Dermo1cre^+^ strain. Il1r^fl/fl^Dermo1cre^−^ mice were used as the control.

### Animal experiments

8–10-week-old male and female mice were implanted with osmotic minipumps (Alzet model 2001) and constant infusion of angiotensin II (1.5 ug/g/day) and phenylephrine (50 ug/g/day). Mice were treated with either an isotype control or an anti-IL-1b neutralizing antibody (Amgen) at 5 mg/kg via intraperitoneal injection every 3 days starting with a pre-treatment injection one day before osmotic minipump implantation and then at days 2 and 5. Mice at day 7 as described in the relevant sections. For the Il1r^fl/fl^Dermo1cre^+^ Ang II/PE experiment 28-day osmotic minipumps (Alzet model 2004) and mice were sacrificed on day 28 for trichrome staining.

### Fibroblast flow cytometry and sorting for single cell RNA seq analysis

After ice cold PBS perfusion, heart tissue from mice were minced with a razor blade on ice and transferred to a 15 mL conical tube containing 3 mL DMEM with 170 uL Collagenase IV (250 U/mL final concentration), 35 uL DNAse1 (60 U/mL), and 75 uL Hyaluronidase (60 U/mL) and incubated at 37 °C for 45 min with agitation. After 45 min, the digestion reaction was quenched with 6 mL of HBB buffer (2% FBS and 0.2% BSA in HBSS), filtered through 40 um filters into a 50 mL conical tube and transferred back into a 15 mL conical tube to obtain tighter pellets. Samples were then spun down at 4 °C, 1200 rpm for 5 min and the supernatant was discarded. Pellet resuspended in 1 mL ACK Lysis buffer (Gibco A10492-01) and incubated at room temperature for 5 min followed by the addition of 9 mL DMEM and centrifugation (4 °C, 5 min, 1200 rpm). Supernatant was discarded and the pellet was resuspended in 2 mL FACS buffer (2% FBS and 2 mM EDTA in calcium/magnesium free PBS) and centrifugation was repeated in above conditions and supernatant aspirated. Samples were then incubated in Pdpn (BioLegend, 127423, clone 8.1.1), Cd140a (Biolegend, 135911, clone APA5), Cd31 (Biolegend, 102409, clone 390), Fap (Creative Biolabs, HPAB-0171-CN-PE), and Cd45 (Biolegend, 103132, clone 30-F11) with a 1:200 dilution for 30 min. A no Fap negative control was used which contained all antibodies except Fap. Solution was washed 3X with FACS buffer following same centrifugation as above and then resuspended in 500 uL of FACS buffer and 1 uL DAPI (BD Biosciences, 564907) and filtered into filter-top FACS tubes. Gating was performed as follows: Pdpn+, singlets, Cd45−/Cd31−, Pdpn+/PDgfra+, and Fap vs Pdgfra gate was used to look at Fap+ fibroblasts. The no Fap antibody negative control was used to draw the Fap gate.

For the fibroblast scRNA-seq experiment, 4 isotype Ang II/PE and 4 anti-IL-1 mAb + Ang II/PE mice FAP+ and FAP− fibroblasts were sorted into 300 uL cell resuspension buffer (0.04% BSA in PBS). Collected cells were centrifuged as above, pooled across mice, and resuspended in collection buffer to a target concentration of 1,000 cells/uL. Cells were counted on a hemocytometer before proceeding with the 10x protocol. cDNA construction and library preparation were performed for 4 libraries (Isotype + Ang II/PE FAP+, Isotype + Ang II/PE FAP−, anti-IL-1b mAb + Ang II/PE FAP+, and anti-IL-1b mAb + Ang II/PE FAP−. cDNA synthesis, library construction, and sequencing were performed using the same protocol as the human CITE-seq data.

### Histology and Immunofluorescence

Flash frozen LV samples were fixed for 24 hr at 4 °C in 10% neutral buffered formalin, washed in 1X PBS, and embedded in paraffin. Paraffin-embedded sections were cut at an 8 um thickness using a microtome. Slides were baked at 65 °C for 1 hr, deparaffinized with serial xylene washes, and rehydrated with ethanol. Then slides underwent methanol treatment (10% MeOH + 3% H_2_O_2_) for 20 min at RT followed by 3X TBS-T washes (5 min each). Antigen retrieval was performed using the AR6 buffer (Ankoya Biosciences, AR600250ML) for 15 min in the microwave and then cooled to room temperature. Tissue sections were marked with a hydrophobic pen and blocked in 10% BSA in TBS-T for 30 min at RT. Slides were then stained with the primary antibody diluted in 10% BSA in TBS-T overnight at 4 °C: FAP (Abcam, ab207178, 1:250) and POSTN (Abcam, ab215199, 1:250). Next, the primary antibody was detected using Opal Polymer HRP Ms + Rb (PerkinElmer Opal Multicolor IHC system). The PerkinElmer Opal Multicolor IHC system was utilized to visualize antibody staining per manufacturer protocol. Slides were imaged using a Zeiss Axioscan Z1 automated slide scanner. Image processing was performed using Zen Blue and Zen Black (Zeiss). For POSTN quantification, the whole heart was circled, and an MFI calculated in sham, isotype + Ang II/PE and anti-IL-1b mAb + Ang II/PE representative sections. For the Il1r^fl/fl^Dermo1cre^+^ mice 28-day Ang II/PE infusion, mice were sacrificed on day 28 and FFPE sections prepared as above. Trichrome staining was performed using Gomori’s Trichrome Stain Kit (ThermoScientific, 87020) and subsequently percent fibrosis was quantified in serial crosss-ections.

For the CCR2 GFP mice, frozen sections were prepared using the following: hearts perfused with cold PBS, placed in 4% PFA overnight at 4 °C, washed with PBS 3X, infiltrated with 30% sucrose overnight at 4 °C, and embedded in OCT and stored in a −80 °C freezer. Prior to staining, 10 um sections were cut using a Leica Cryostat, washed with PBS, incubated with 0.25% Triton-X in PBS at RT for 5 min, blocked in 5% BSA/PBS for 1hr at RT, stained with primary antibody: anti-GFP (Abcam, ab13970) and IL-1b (R&D systems, AF-401-NA, 1:50) co-stained with an appropriate secondary antibody, and mounted with DAPI fluoroshield (R&D, F6057). Slides were then imaged on a Zeiss confocal microscopy and representative images included.

### RT- PCR

RNA was extracted from flash frozen mouse apex using the Qiagen RNeasy Plus Mini Kit. RNA concentration was then measured using a nanodrop spectrophotometer and cDNA synthesis was performed using the HighCapacity RNA to cDNA synthesis kit (Applied Biosystems). Quantitative real time PCR reactions were prepared with sequence-specific primers with PowerUP^™^ Syber Green Master mix (Thermo Fisher Scientific) in a 20 uL volume. Real time PCR was performed using QuantStudio3 (Thermo Fisher Scientific). mRNA expression was normalized to 36B4. The Postn primer was purchased from ADT (Fwd: 5’-GGTGCCCTAGAAAGGATCATGG-3’; Rev: 5’-CAGAGCACTGGAGGGTATTTAG-3’).

## Supplementary Material

Supplement 1

## Data Availability

Raw sequencing files can be found on the Gene Expression Omnibus super series (GSE218392). Processed data is available upon request from the authors.
