# Supplementary material for: Targeting Immune-Fibroblast Crosstalk in Myocardial Infarction and Cardiac Fibrosis
Source: Res Sq. 2023 Jan 26:rs.3.rs-2402606. Preprint. [Version 1] doi: 10.21203/rs.3.rs-2402606/v1 (PMC9900986; doi:10.21203/rs.3.rs-2402606/v1)
Supplement: Supplement 1 [file NIHPPrs2402606v1-supplement-1.pdf]

Supplementary Figure 1

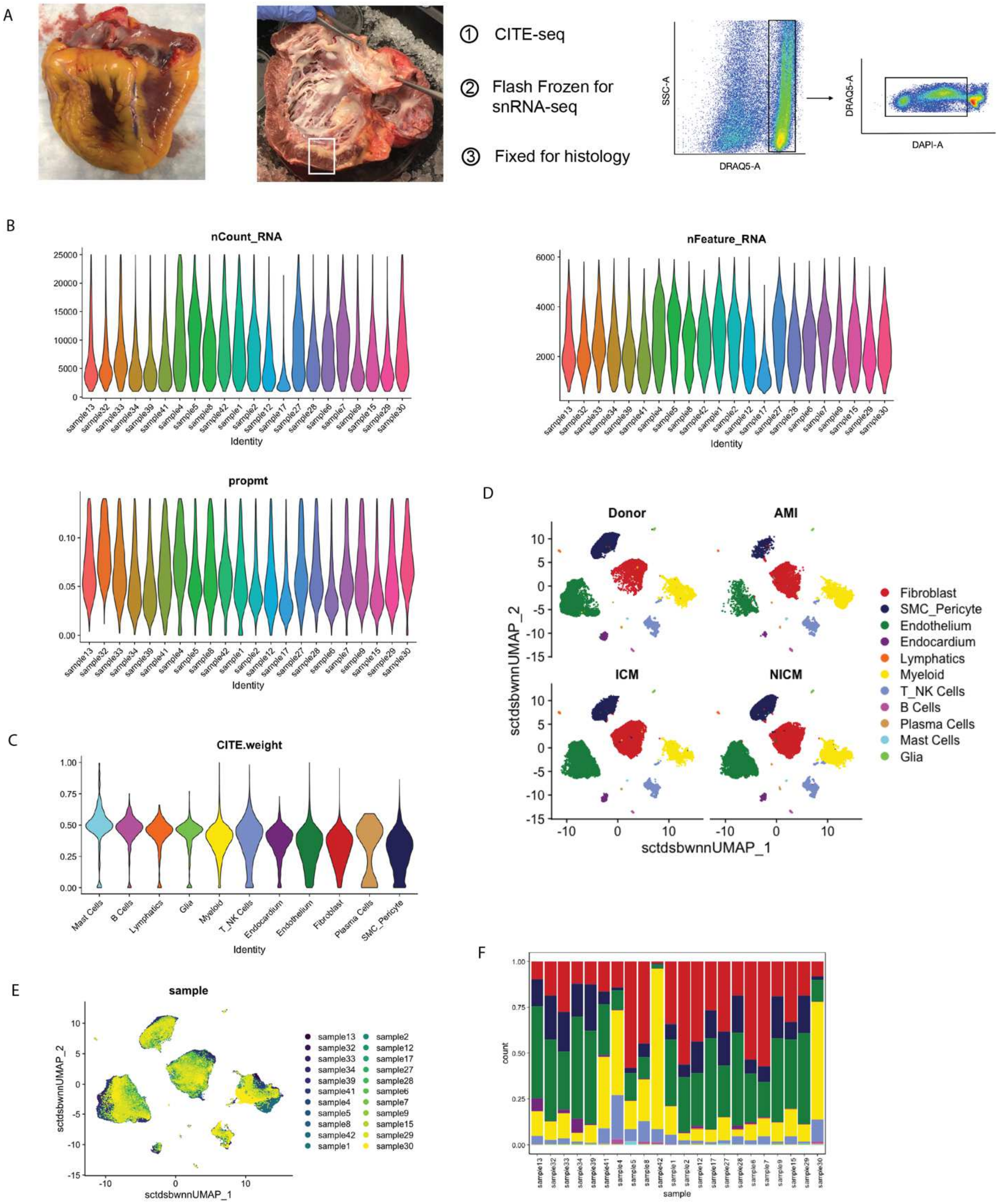

Supplementary Figure 1. (A) Explanted heart and dissected region boxed used for CITE-seq, snRNA-seq, histological analysis with flow cytometry panel to sort live cells. (B) QC metrics post filtering. (C) CITE-seq protein assay weights used for WNN clustering. (D) Global integrated UMAP split by 4 conditions. (E) Integrated global UMAP colored by patient sample. (F) Cell type composition split by each sample.

Supplementary Figure 2

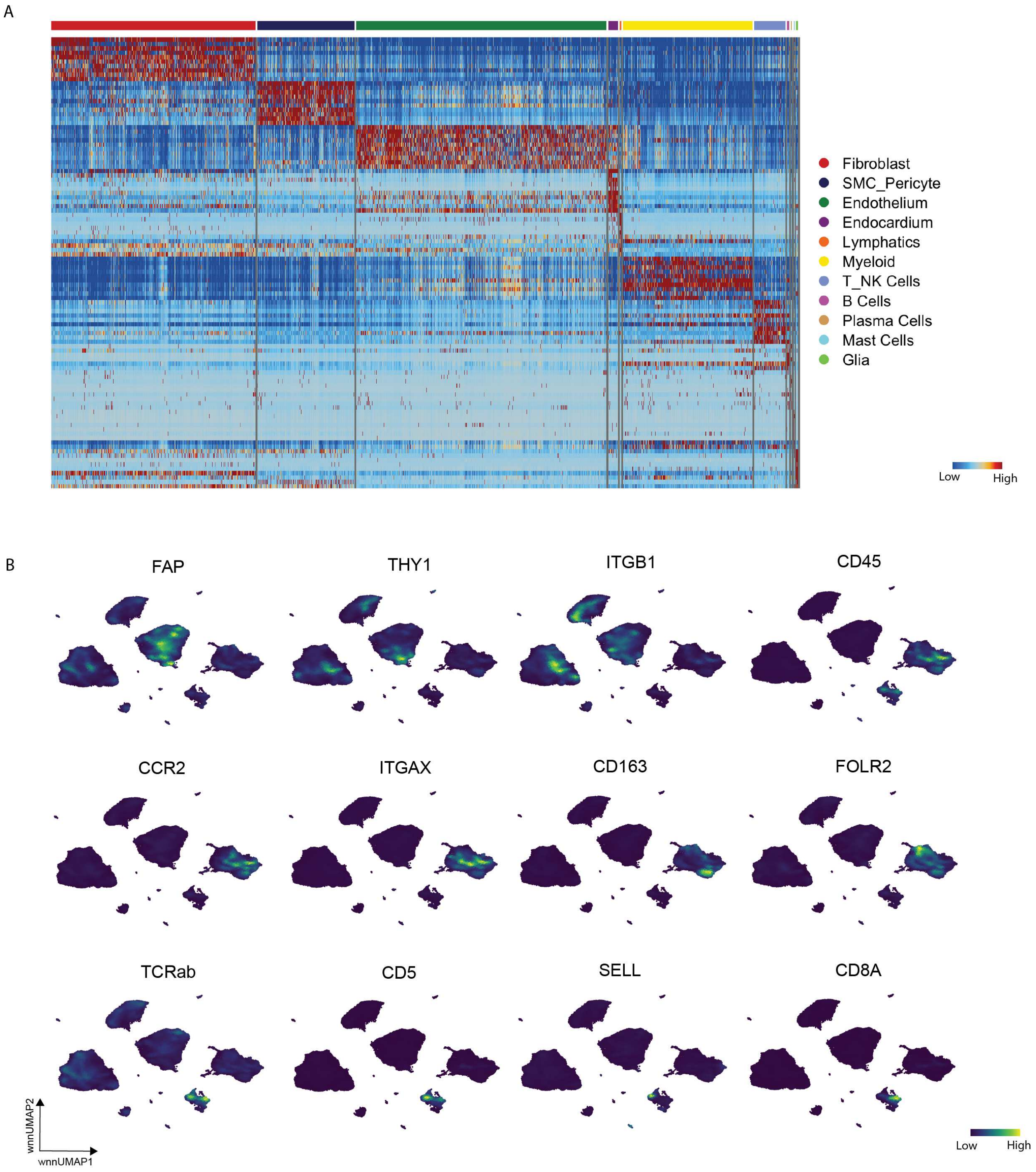

Supplementary Figure 2. (A) Heatmap of top marker genes for each cell type in global UMAP. (B) Protein expression as a density plot for top CITE-seq protein markers in different cell types.

Supplementary Figure 3

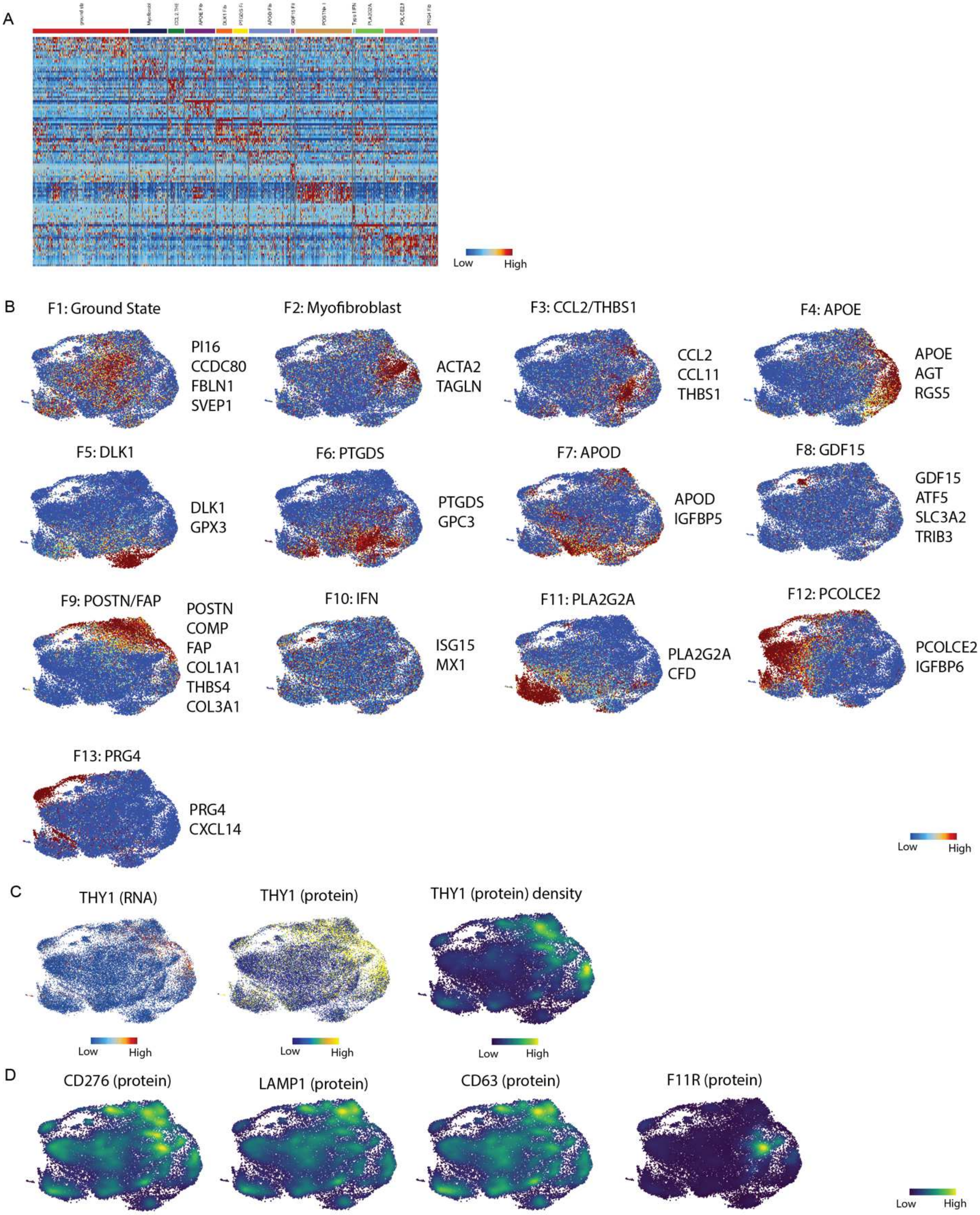

Supplementary Figure 3. (A) Heatmap of top marker genes for fibroblast cell states. (B) Gene set z-scores for fibroblast cell states plotted in UMAP embedding. (C) THY1 RNA, protein, and protein density plot in fibroblast UMAP space. (D) Density plots for differentially expressed protein markers in fibroblast UMAP space.

Supplementary Figure 4

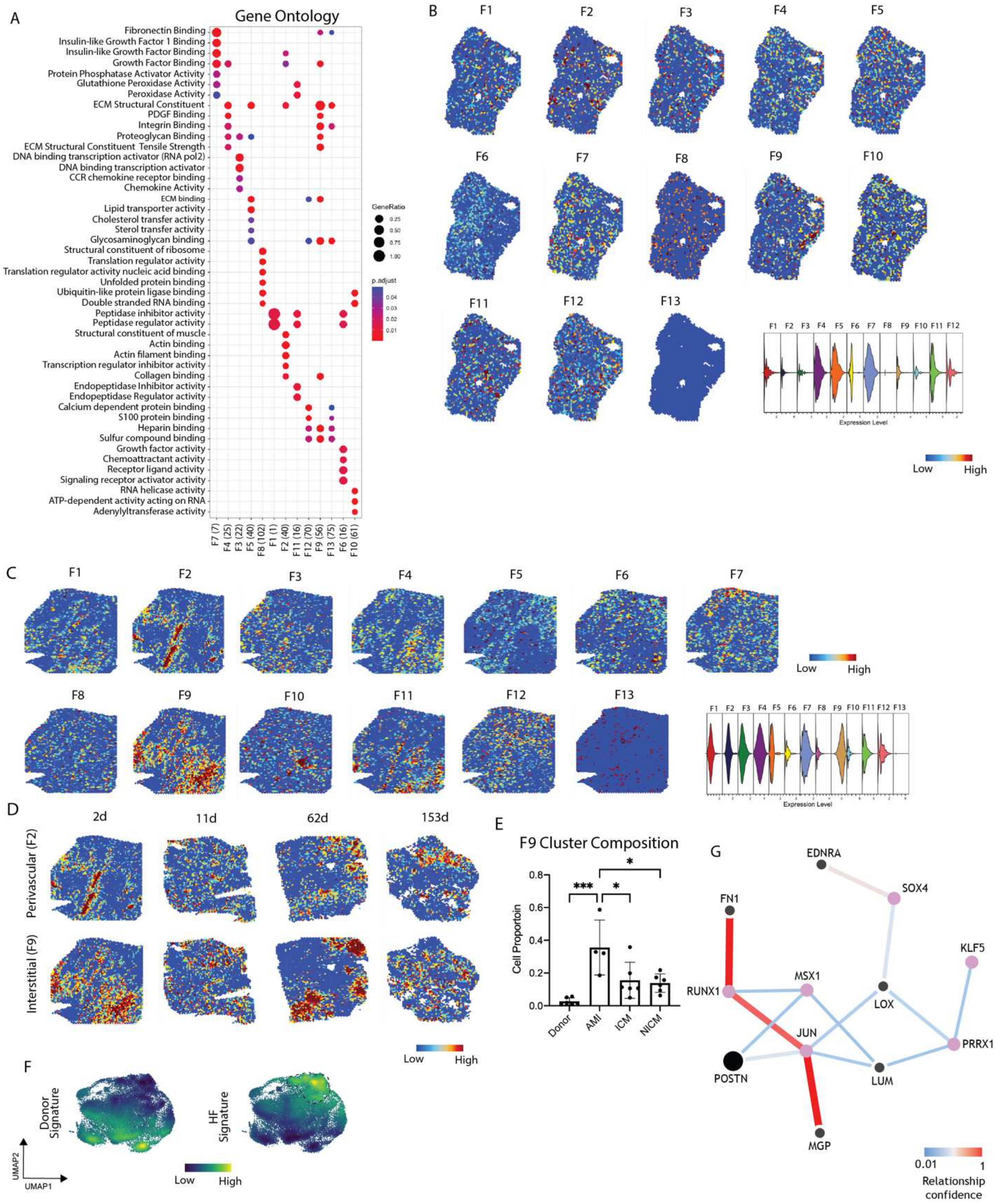

Supplementary Figure 4. (A) GO analysis from clusterProfiler for fibroblast cell states. Fibroblast cell state gene set scores in a spatial transcriptomics (B) donor and (C) AMI sample with associated violin plot. (D) F2 and F9 gene set score plotted in spatial transcriptomics data across acute MI and ICM sections. (E) F9: FAP/POSTN cluster composition across four groups. One-way ANOVA test with multiple comparisons: donor vs acute MI (***P = 0.0002), acute MI vs ICM (*P = 0.0207), and acute MI vs NICM (*P = 0.0115). (F) Gene set signature kernel density embedding plot for donor (top) and HF (bottom) – genes are derived from pseudobulk differential expression analysis between donor and HF. Statistically significant genes (adjusted p-value < 0.05, log2FC > 0.58, and base mean expression > 500). (G) Transcriptional regulation interaction between *POSTN*, *EDNRA,* and *MEOX1* using PathwayNet (http://pathwaynet.princeton.edu).

Supplementary Figure 5

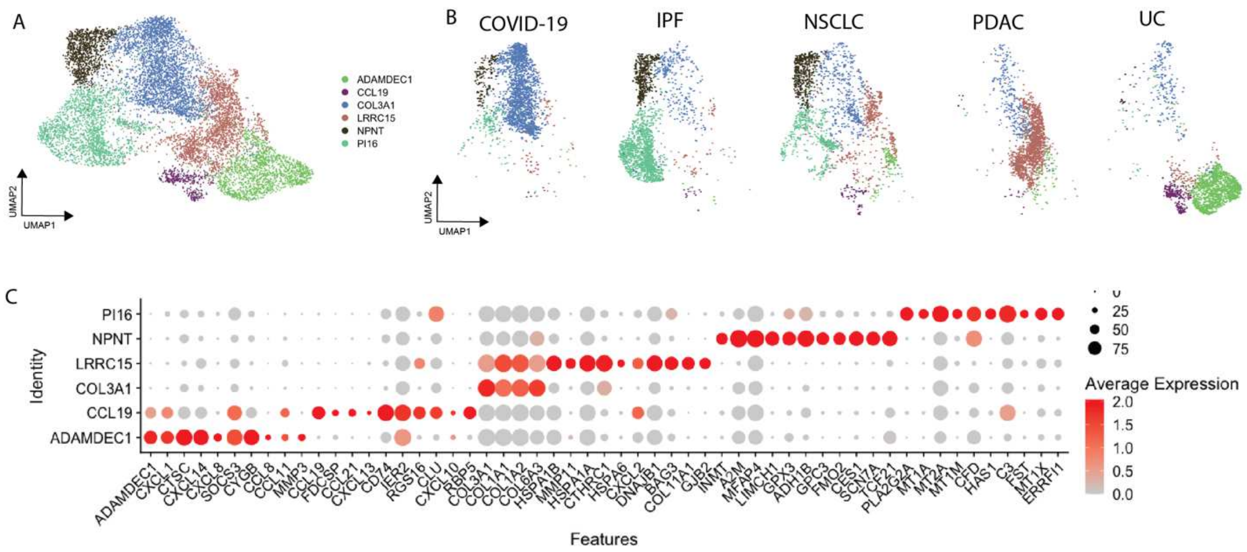

Supplementary Figure 5. (A) Integrated UMAP of perturbated pathological fibroblasts and (B) split by disease category. (C) Dotplot of marker genes for clusters in (A).

Supplementary Figure 6

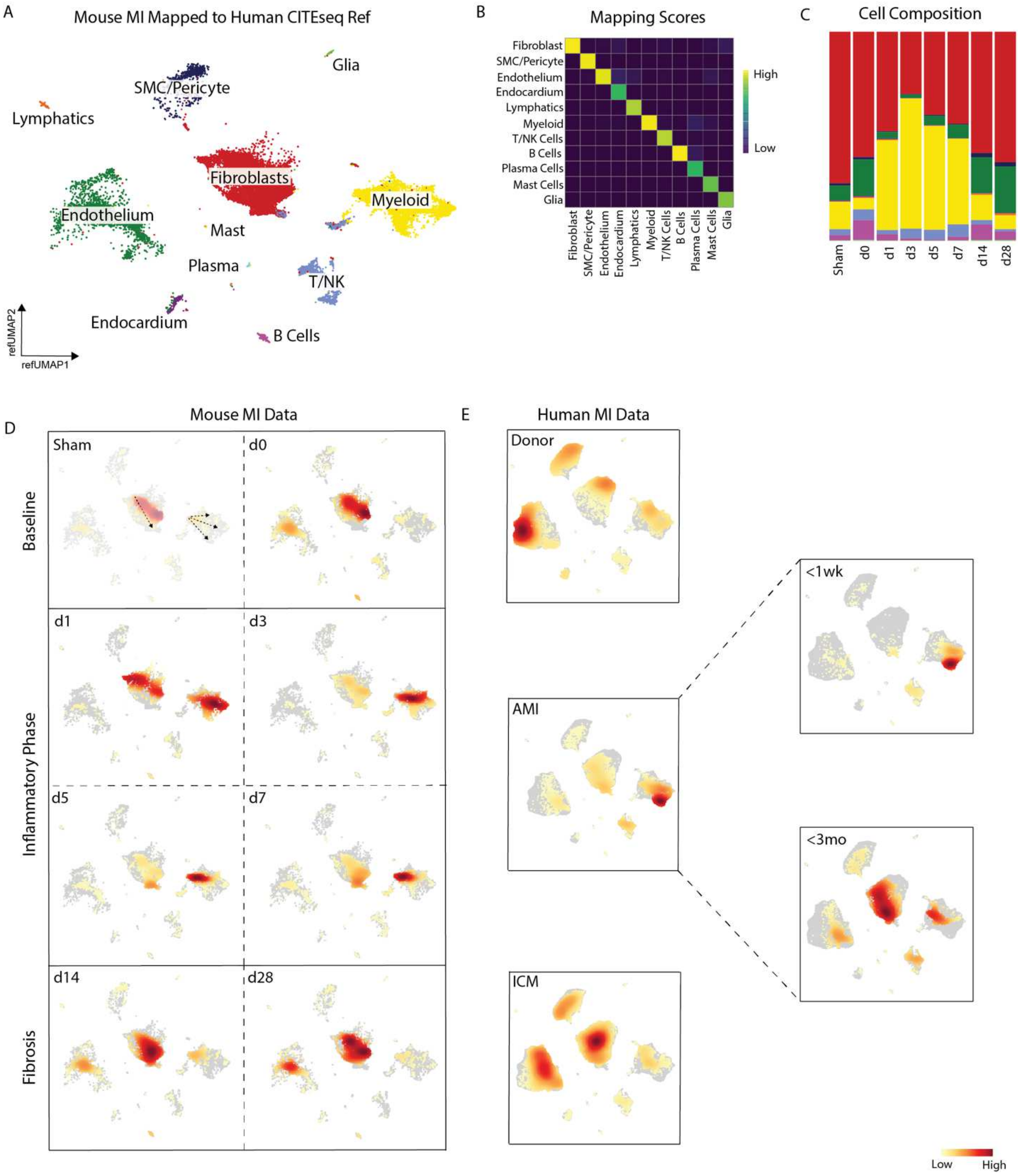

Supplementary Figure 6. (A) Mouse MI data mapped onto human CITE-seq global object. (B) Seurat reference mapping scores for different cell types. (C) Cell composition from imputed annotations split by time point. (D) Gaussian kernel density embedding plots in mouse MI data split by condition and biological phase and (E) human MI data grouped by MI category.

Supplementary Figure 7

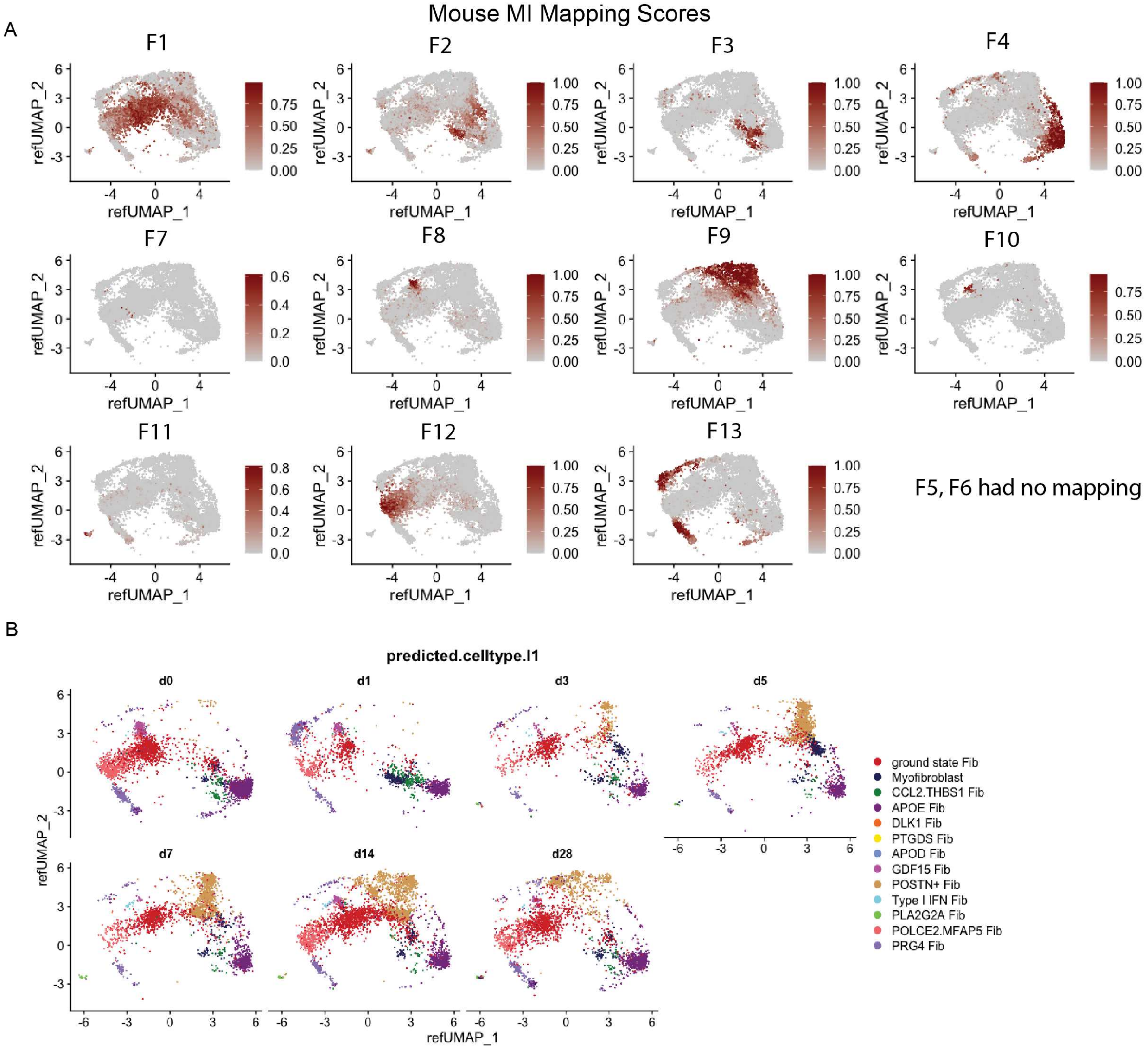

Supplementary Figure 7. (A) Reference mapping scores for mouse MI fibroblasts onto human heart CITE-seq fibroblast UMAP embedding. (B) Reference mapped data split by MI time point in human space.

Supplementary Figure 8

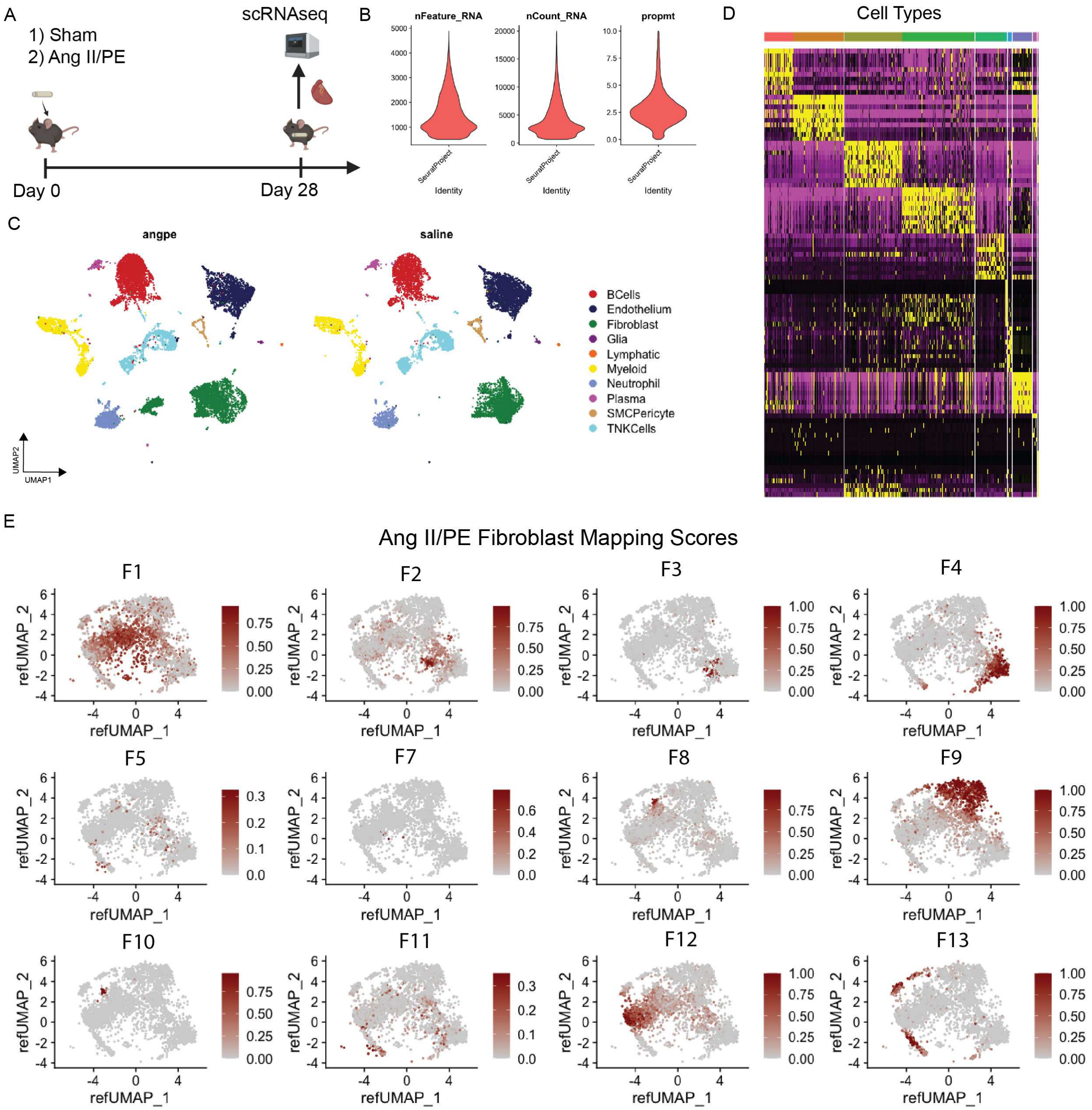

Supplementary Figure 8. (A) Experimental design for Ang II/PE 28-day pumps for sequencing. (B) QC metrics post filtering. (C) Integrated global UMAP split by sham and Ang II/PE at day 28. (D) Heatmap of top marker genes for clusters from (C). (E) Reference mapping scores for mouse Ang II/PE and sham d28 fibroblasts onto human heart CITE-seq fibroblast UMAP embedding.

Supplementary Figure 9

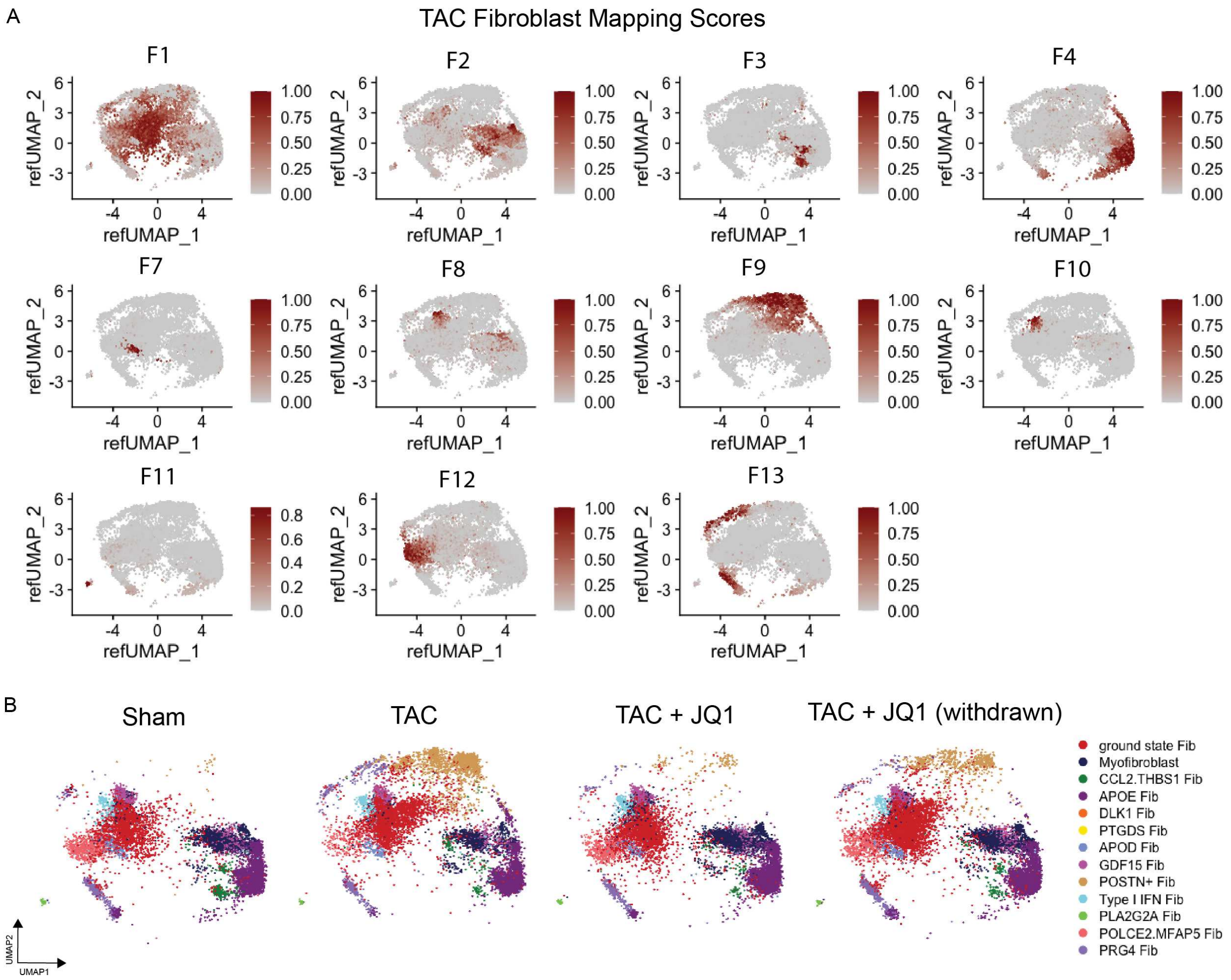

Supplementary Figure 9. (A) Reference mapping scores for mouse TAC fibroblasts onto human heart CITE-seq fibroblast UMAP embedding. (B) Reference mapped data split by sham, TAC, TAC + JQ1 treatment, and TAC + JQ1 withdrawn in human space.

Supplementary Figure 10

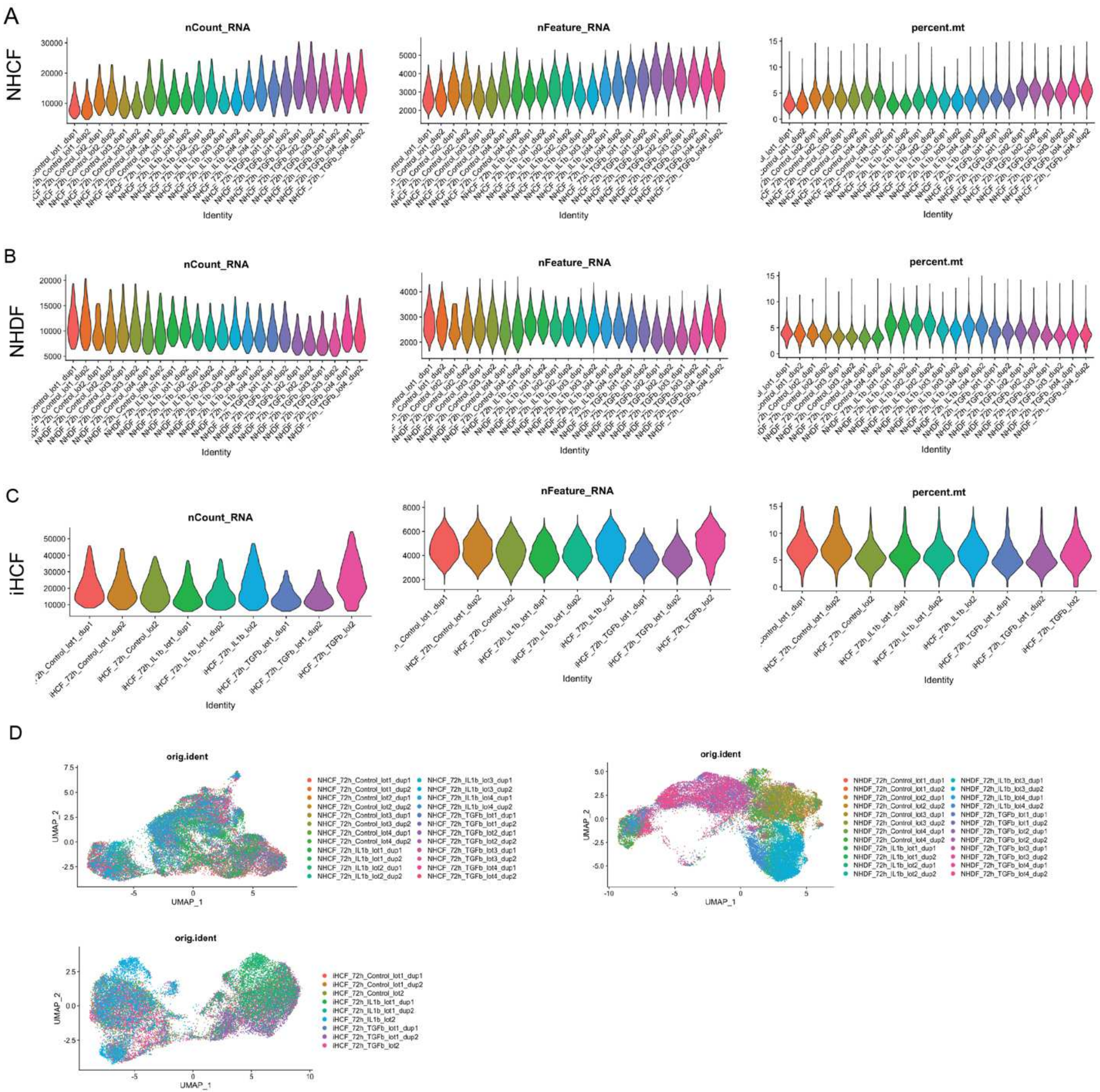

Supplementary Figure 10. Quality control metrics post filtering grouped by each biological replicate for each experimental condition (vehicle, TGF-b, and IL-1b) in (A) NHCF, (B) NHDF, and (C) iHCF. (D) Integrated data from 3 cell lines in a UMAP embedding colored by biological replicate for each experimental condition.

Supplementary Figure 11

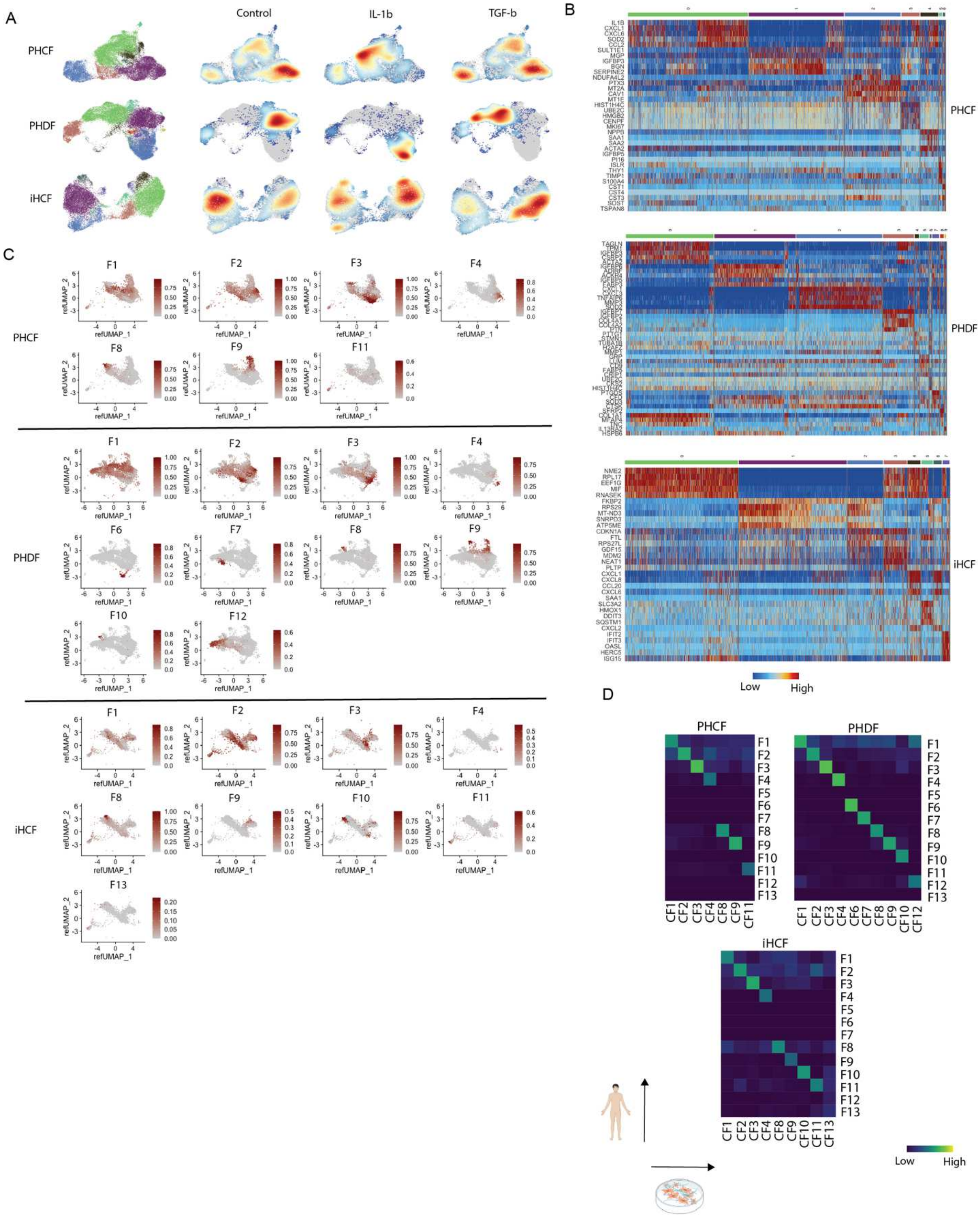

Supplementary Figure 11. (A) UMAP embedding with separate clustering in each cell line with density plots split by experimental condition. (B) Heatmap of marker genes for each cell line for clusters in (A). (C) Reference mapping scores for *in vitro* fibroblasts onto human heart CITE-seq fibroblast UMAP embedding. (D) Heatmap of mapping scores with *in vitro* clusters on x-axis and human heart CITE-seq fibroblasts on y-axis. (E) Human, *in vivo*, and *in vitro* fibroblasts integrated in a common UMAP space colored by category. (F) Phylogeny tree clustering of categories from integrated data in (E).

Supplementary Figure 12

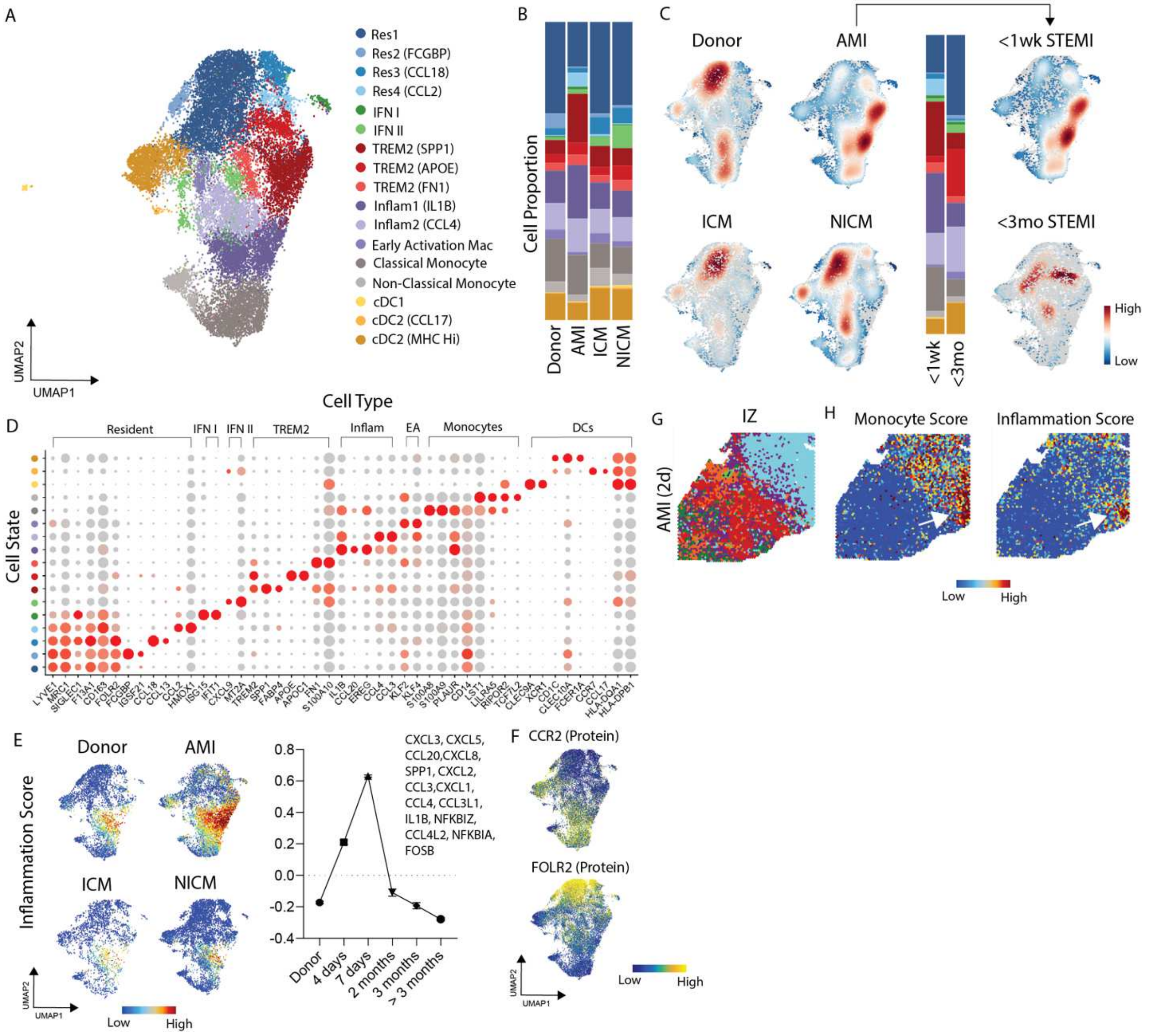

Supplementary Figure 12. (A) UMAP embedding plot of myeloid cells with annotated cell states. (B) Myeloid cell state composition across four groups. (C) Gaussian kernel density estimation of cells across four groups (left) and split by MI time in acute MI patients with corresponding cell state composition. (D) Dot Plot of marker genes for macrophages cell states (y-axis) and grouped by cell type (x-asis). (E) Inflammation gene set score split across 4 groups and (g) grouped from time post-MI. (F) CCR2 and FOLR2 (protein) expression in UMAP embedding. (G) Spatial transcriptomic acute MI (2-day post-MI) infarct zone (IZ) sample with label transferred annotations from snRNA-seq reference map. (H) Monocyte gene set score mapped into space (left) and inflammation score (right).

Supplementary Figure 13

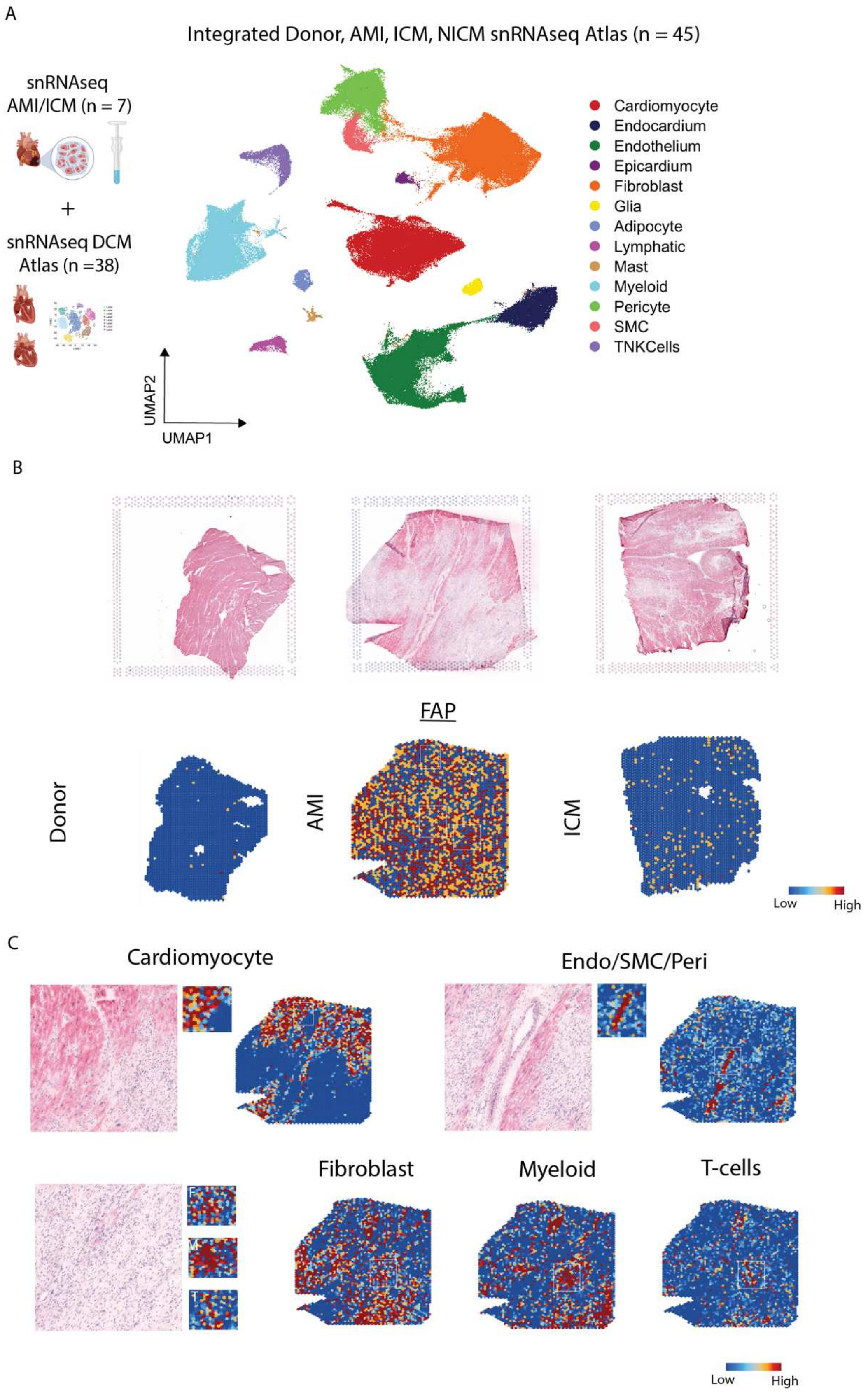

Supplementary Figure 13. (A) UMAP embedding of integrated single nuclei RNA-seq data from donor, acute MI, ICM, and NICM. (B) HE spatial transcriptomics sections and corresponding FAP RNA expression in space. (C) Gene set signatures for cardiomyocytes, SMC/endothelium/pericytes, fibroblasts, myeloid cells, and T cells from clusters in (A) plotted in an acute MI sample.

Supplementary Figure 14

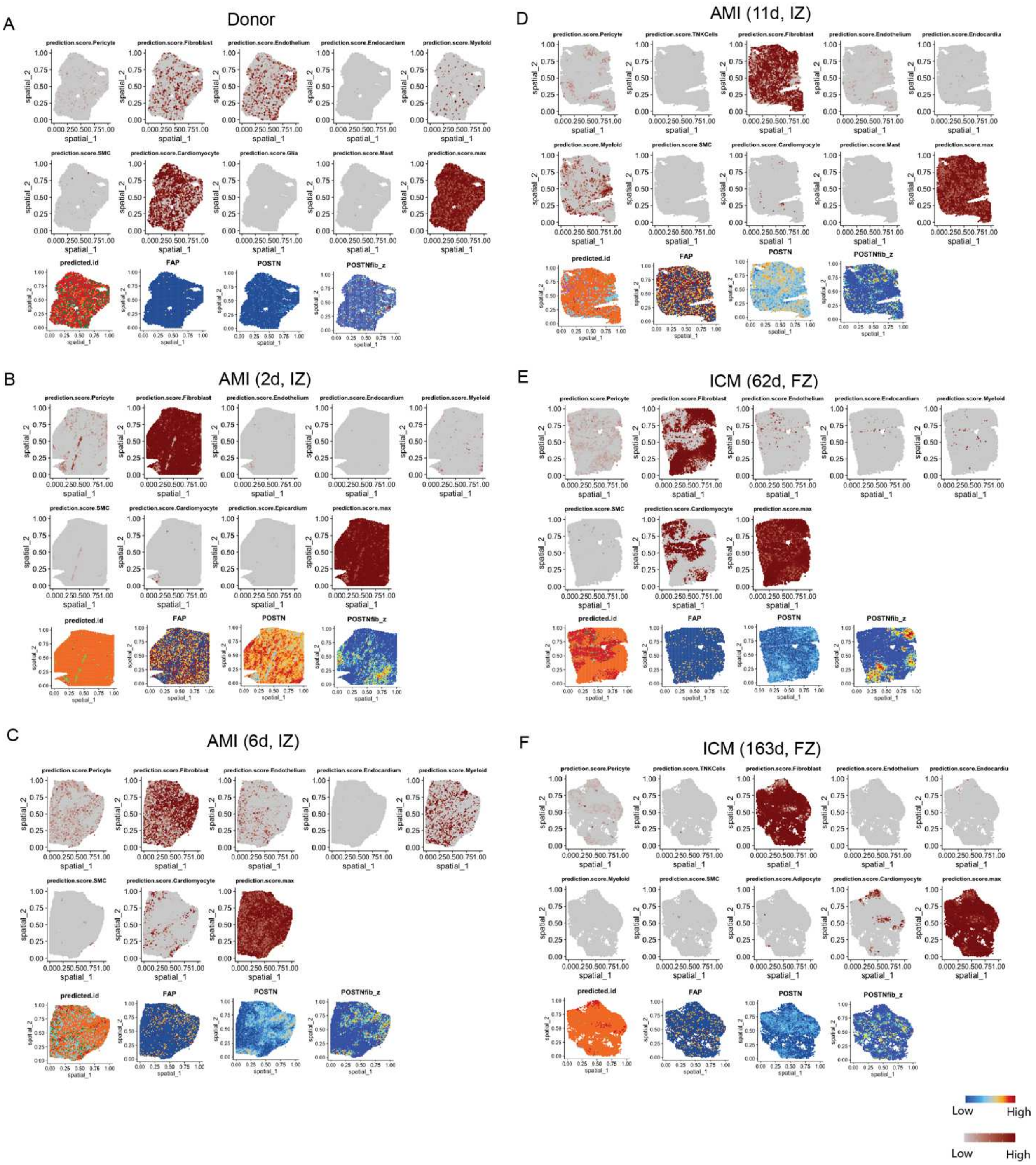

Supplementary Figure 14. Human snRNA-seq reference map imputed prediction scores, annotations, FAP expression, POSTN expression, and F9 fibroblast gene set score in (A) donor, (B) 2d acute MI IZ, (C) 6d acute MI IZ, (D) 11d acute MI IZ, (E) 62d ICM, FZ, and (F) 163d ICM FZ sptial transcriptomic sections.

Supplementary Figure 15

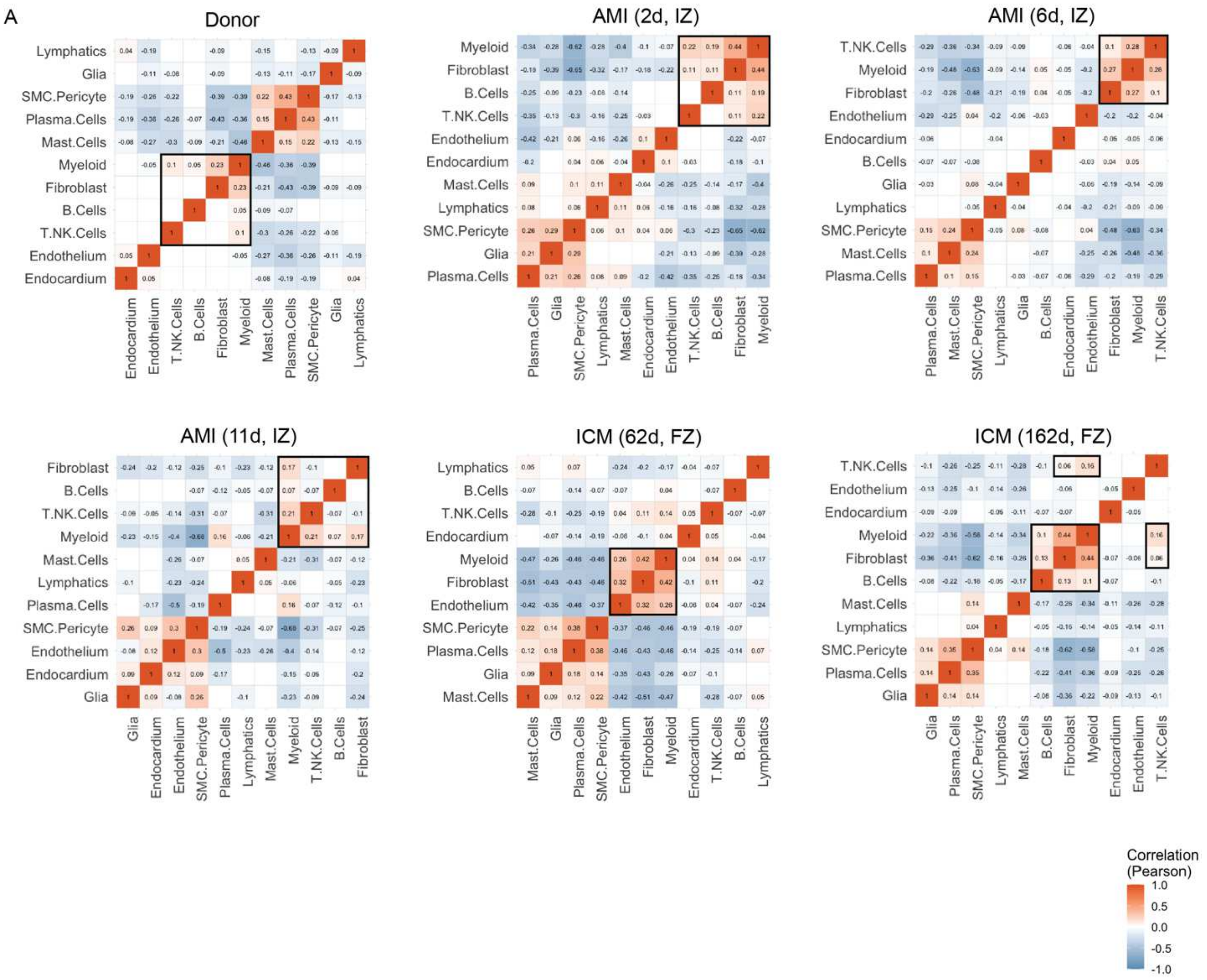

Supplementary Figure 15. (A) SPOTlight derived cell spot deconvolution Pearson correlation coefficients for cell neighborhoods in donor, acute MI and ICM spatial transcriptomic sections.

Supplementary Figure 16

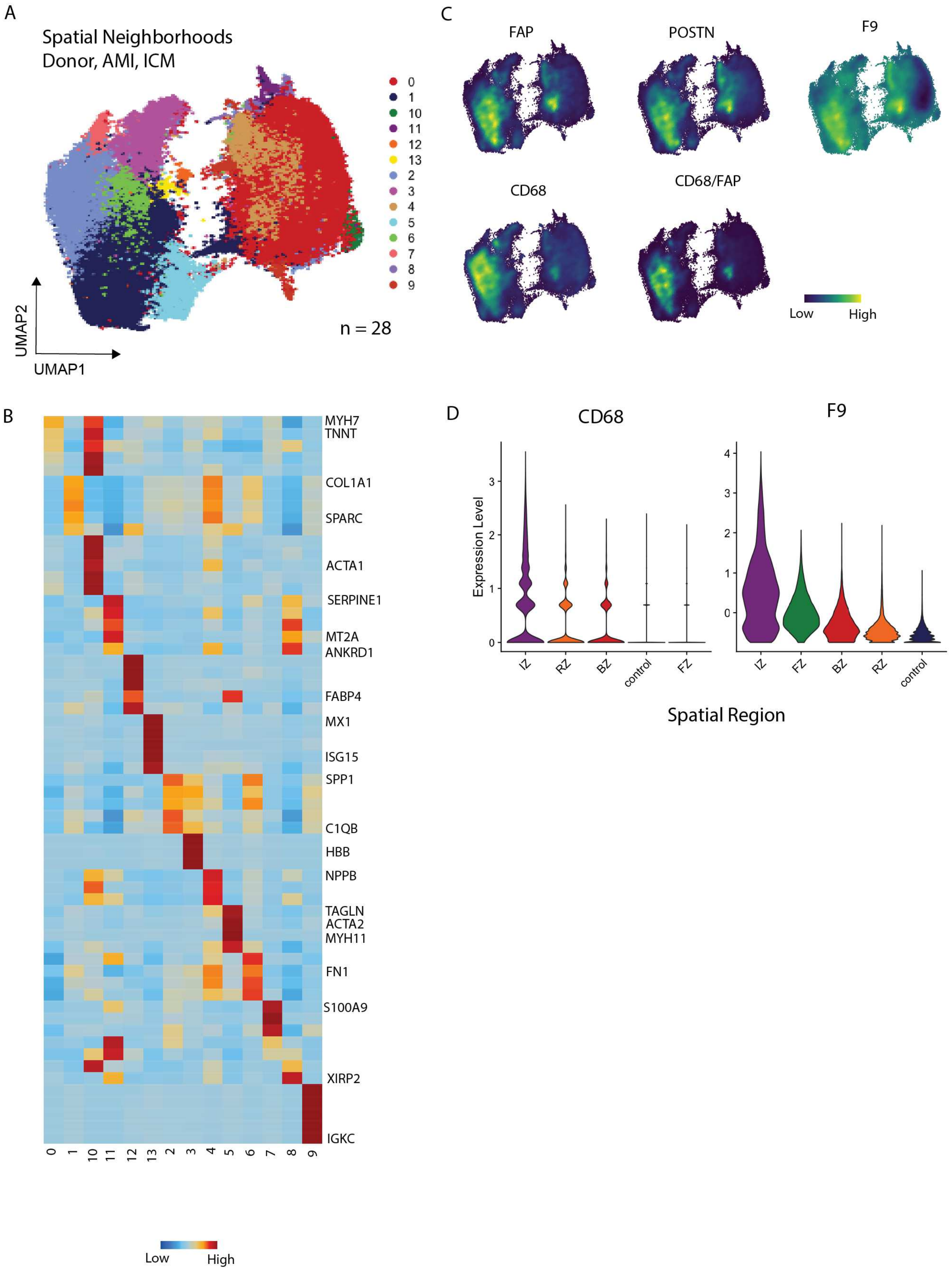

Supplementary Figure 16. (A) Integrated UMAP embedding of n=28 spatial transcriptomic samples spatial spots. (B) Heatmap of average expression for top marker genes for spatial niches from (A). (C) FAP, POSTN, CD68 expression, CD68/FAP joint, and F9 gene set score density expression plots in integrated spatial UMAP embedding. (D) CD68 expression and F9 fibroblast gene set score violin plot split by regions.

Supplementary Figure 17

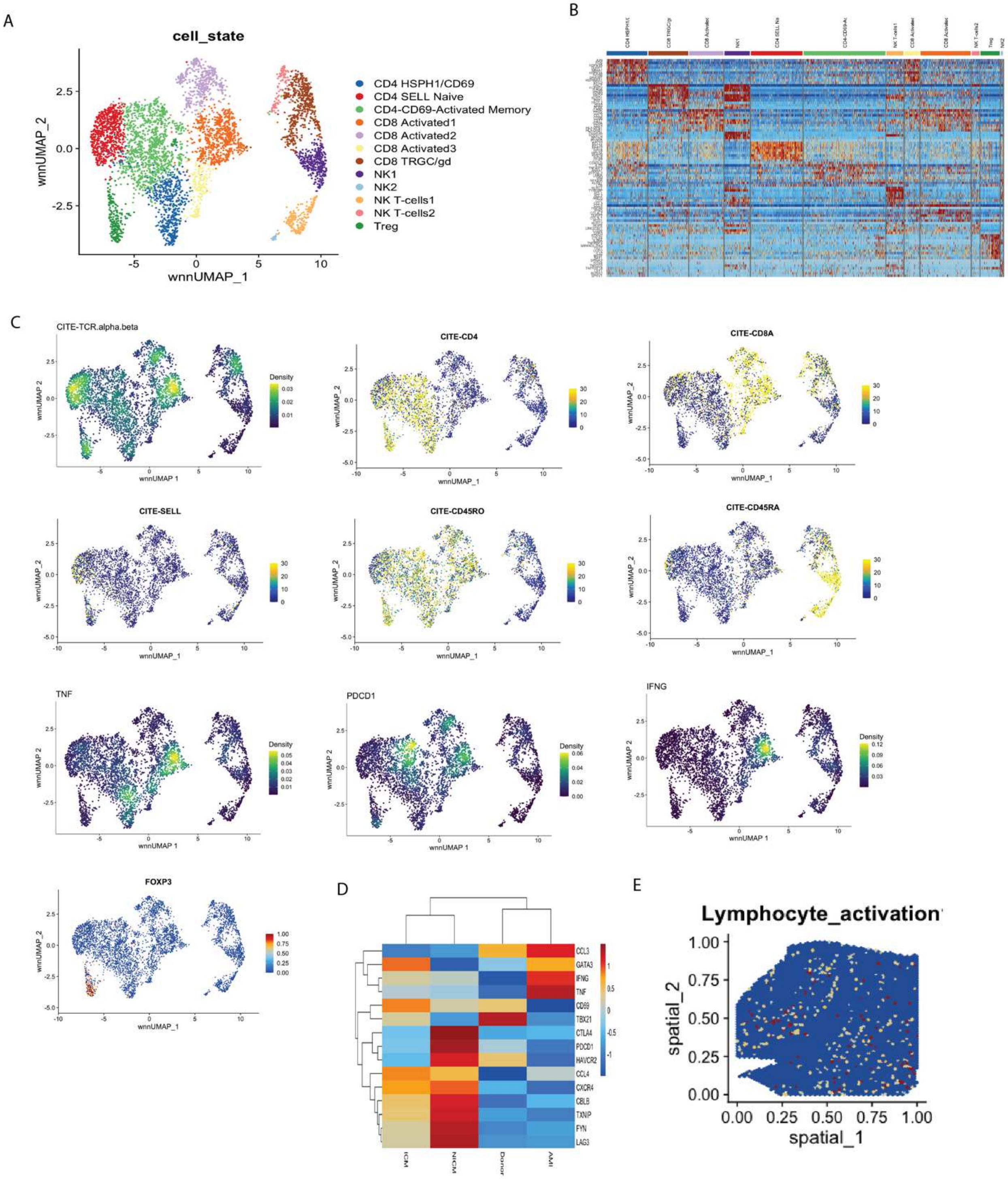

Supplementary Figure 17. (A) T cell states in WNN embedding space. (B) Top RNA markers for T cell states from (A). (C) Density, protein, and RNA plots for differentially expressed and key T cell markers. (D) RNA expression heatmap of key T cell genes (in different phenotypes) grouped by condition. (E) Lymphocyte activation signature plotted in AMI IZ spatial transcriptomic section.

Supplementary Figure 18

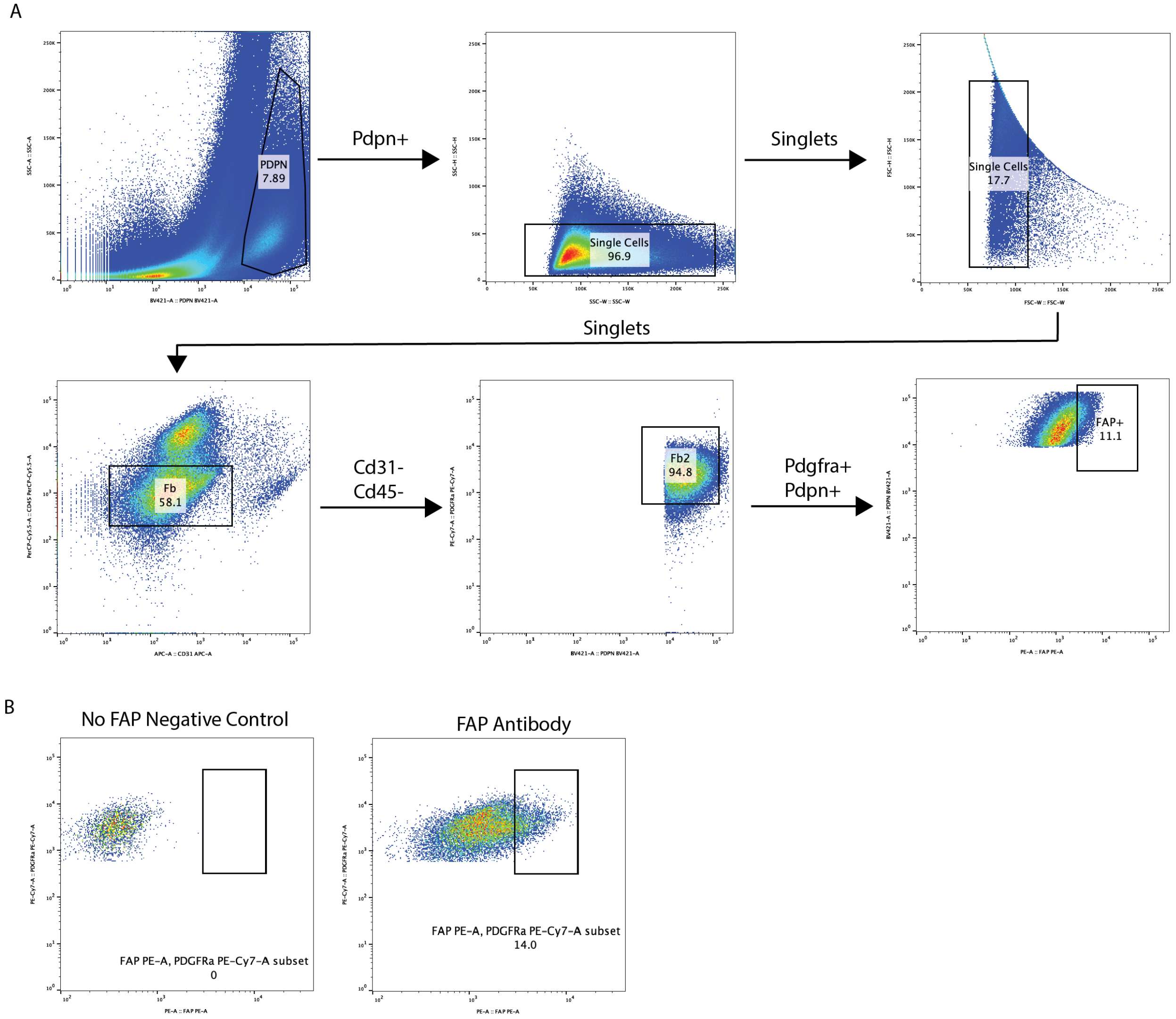

Supplementary Figure 18. (A) Flow cytometry gating scheme for fibroblasts. (B) FAP+ gate construction with a no antibody-stained negative control.

Supplementary Figure 19

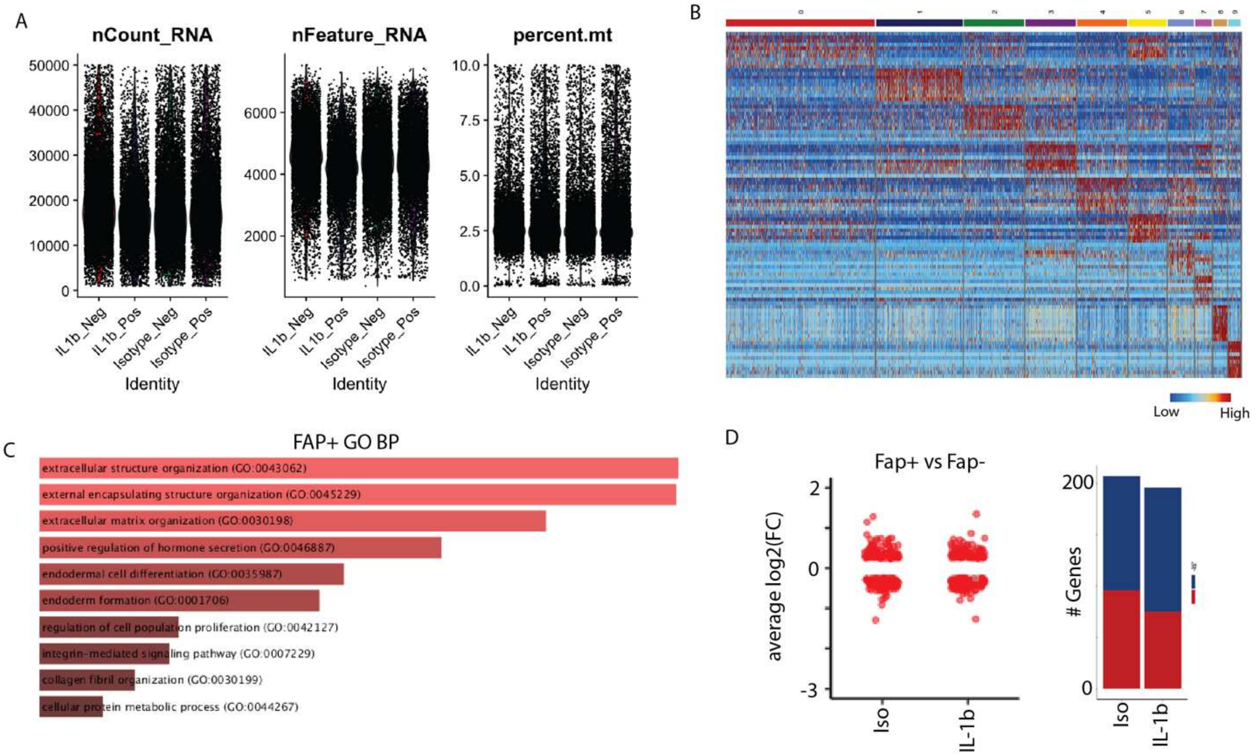

Supplementary Figure 19. (A) QC metrics grouped by experimental condition for in vivo sorted FAP+/- fibroblasts at day 7 in Ang II/PE model with isotype/anti-IL-1b mAb treatment. (B) Heatmap of top marker genes for clusters in Fig. 7B. (C) Top GO BP pathways enriched in FAP+ fibroblasts relative to FAP- fibroblasts. (D) DE analysis between FAP+ and FAP- fibroblasts split by isotype and anti-IL-1b mAb groups dot plot (left) and number of genes up/down quantified (right).

Supplementary Figure 20

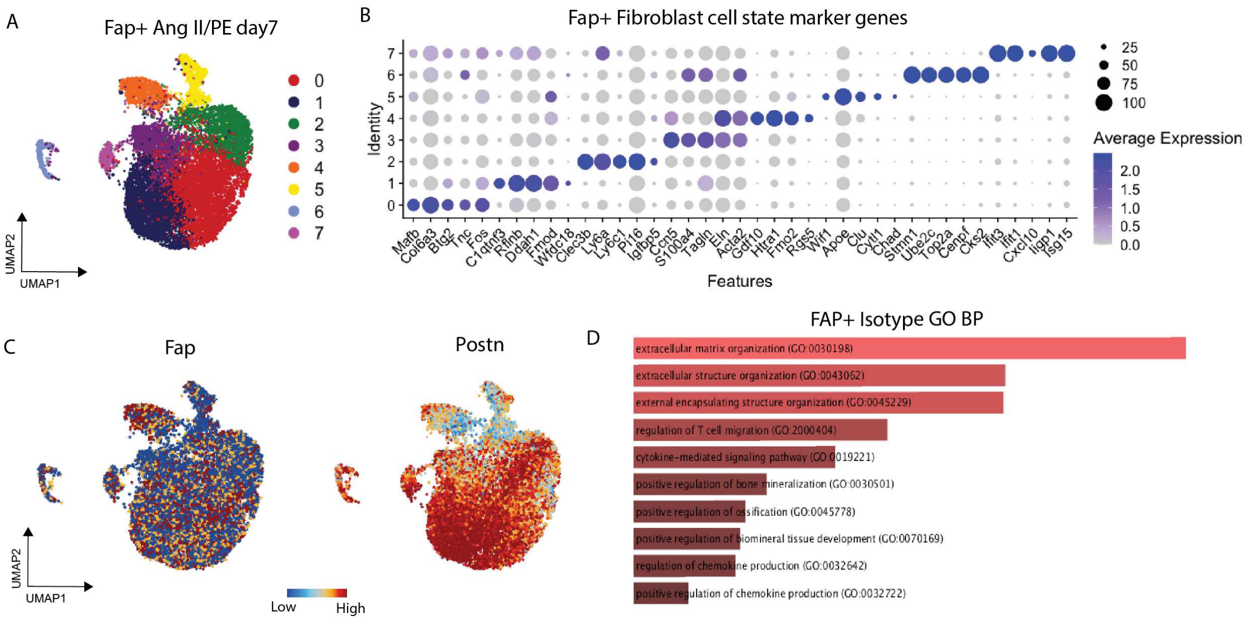

Supplementary Figure 20. (A) Integrated UMAP with cub-clustering of FAP+ fibroblasts at day 7 in Ang II/PE. (B) DotPlot of top marker genes for FAP+ cell states from (A). (C) Fap and Postn expression in UMAP embedding. (D) GO BP pathways enriched in isotype treated FAP+ fibroblasts relative to FAP+ anti-IL-1b mAb treated mice.

Supplementary Figure 21

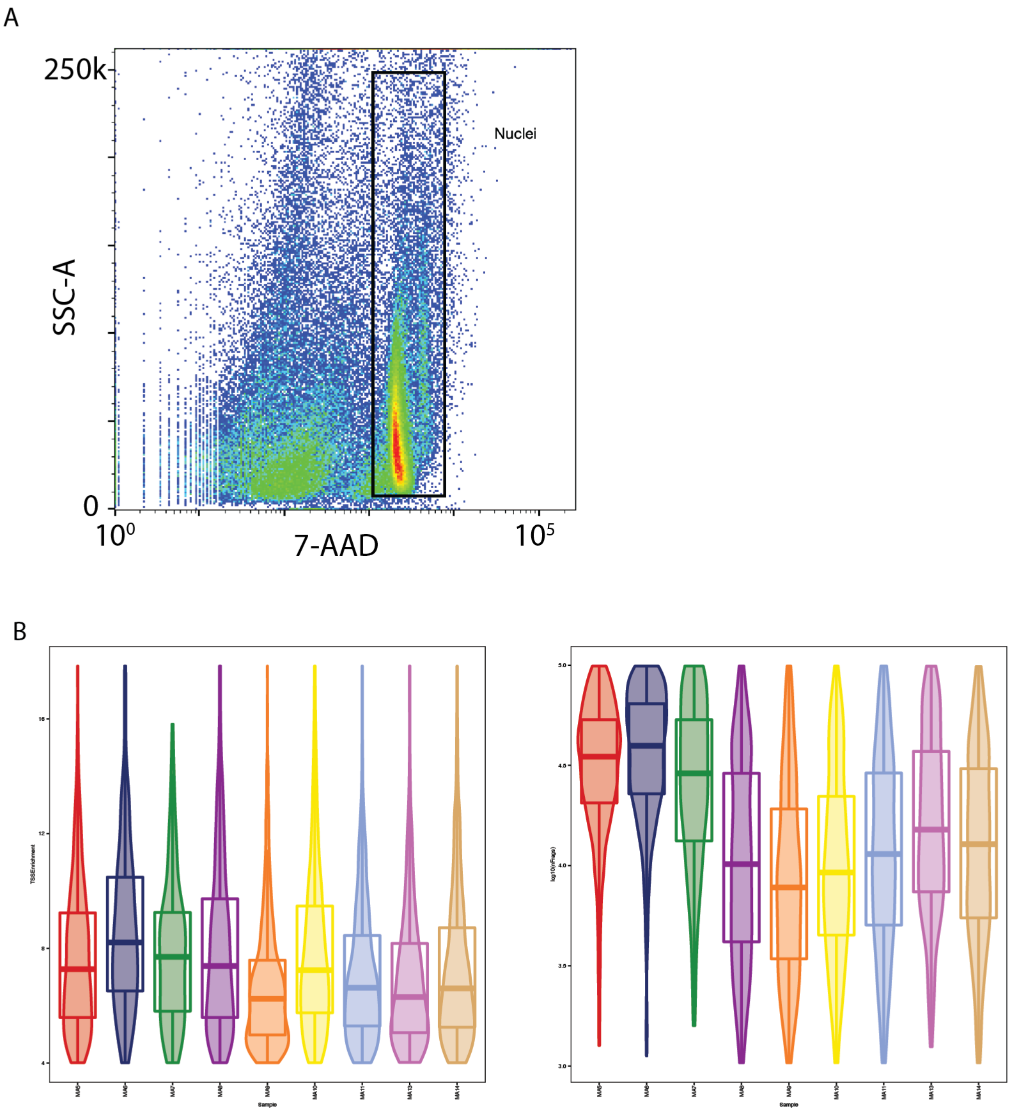

Supplementary Figure 21. (A) Flow cytometry gating scheme to isolate nuclei using 7-AAD. (B) Multiome samples quality control metrics post QC and doublet removal box-violin plot split by sample; TSS enrichment (left) and log10 (nFrags) calculated in ArchR.

Supplementary Files

This is a list of supplementary files associated with this preprint. Click to download.

- table1.csv
